# Probing the Separation Distance between Biological Nanoparticles and Cell Membrane Mimics Using Neutron Reflectometry with Sub-Nanometer Accuracy

**DOI:** 10.1021/jacs.2c08456

**Published:** 2022-11-03

**Authors:** Antonius Armanious, Yuri Gerelli, Samantha Micciulla, Hudson P. Pace, Rebecca J. L. Welbourn, Mattias Sjöberg, Björn Agnarsson, Fredrik Höök

**Affiliations:** † Department of Physics, Chalmers University of Technology, 41296Gothenburg, Sweden; ‡ Institut Max von Laue-Paul Langevin (ILL), 38042Grenoble, France; § Department of Life and Environmental Sciences, Università Politecnica delle Marche, 60131Ancona, Italy; ∥ ISIS Facility, STFC, Rutherford Appleton Laboratory, Chilton, Didcot, OxonOX11 0QX, United Kingdom

## Abstract

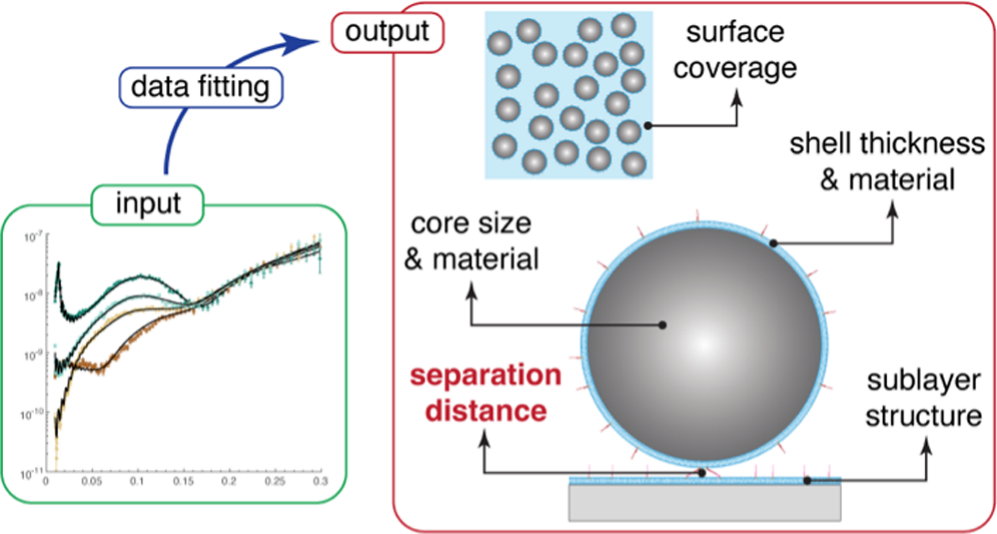

Nanoparticle interactions with cellular membranes are controlled by molecular recognition reactions and regulate a multitude of biological processes, including virus infections, biological nanoparticle-mediated cellular communication, and drug delivery applications. Aided by the design of various supported cell membrane mimics, multiple methods have been employed to investigate these types of interactions, revealing information on nanoparticle coverage, interaction kinetics, as well as binding strength; however, precise quantification of the separation distance across which these delicate interactions occur remains elusive. Here, we demonstrate that carefully designed neutron reflectometry (NR) experiments followed by an attentive selection and application of suitable theoretical models offer a means to quantify the distance separating biological nanoparticles from a supported lipid bilayer (SLB) with sub-nanometer precision. The distance between the nanoparticles and SLBs was tuned by exploiting either direct adsorption or specific binding using DNA tethers with different conformations, revealing separation distances of around 1, 3, and 7 nm with nanometric accuracy. We also show that NR provides precise information on nanoparticle coverage, size distribution, material composition, and potential structural changes in the underlying planar SLB induced upon nanoparticle binding. The precision with which these parameters could be quantified should pave an attractive path for investigations of the interactions between nanoparticles and interfaces at length scales and resolutions that were previously inaccessible. This thus makes it possible to, for example, gain an in-depth understanding of the molecular recognition reactions of inorganic and biological nanoparticles with cellular membranes.

## Introduction

Characterization of nanoparticle interactions that occur within nanometer distances to cellular membranes is crucial for an in-depth understanding of a multitude of biological processes, including neuronal signaling, viral infection, exosome-mediated intracellular communication, nanoparticle-assisted drug and vaccine delivery, as well as nanotoxicological effects. Methods to quantify the molecular length scales over which these types of interactions occur in cellular systems are few and essentially restricted to cryogenic transmission electron microscopy (cryo-TEM),^1−3^ fluorescence resonance energy transfer (FRET),^4−6^ and super-resolution optical microscopy approaches.^7−11^ However, the complex biomolecular content and topological architecture of cells complicate the analysis, particularly the determination of the absolute distances at which biomolecular interactions occur. This, in turn, motivated intensive efforts devoted to the design of reductionistic mimics of cell membranes.^12−28^

Besides offering the opportunity to deliberately define the molecular (lipid, protein, and glycan) composition of the membrane,^20,29−33^ surface-supported artificial cell membranes have also enabled a broader arsenal of advanced analytical tools to be employed. In particular, the application of tools such as surface plasmon resonance (SPR),^34,35^ quartz crystal microbalance (QCM),^36−42^ scanning probe microscopy (SPM),^43,44^ total internal reflection fluorescence (TIRF) microscopy,^38,45−48^ and surface-sensitive optical scattering techniques,^49,50^ advanced the understanding of how nanoparticles interact with cellular membranes, offering key insights on, for example, interaction kinetics,^35,37,38,48,51^ the dependence of lateral nanoparticle diffusivity on the number of molecular contact points,^47,48,52^ as well as membrane fusion of DNA-tethered lipid vesicles,^53,54^ viruses,^55,56^ and SNARE-associated synaptic vesicles.^57^ These methods, alone or in combination, can also provide quantitative information on the mass and number of bound nanoparticles,^40,45,50^ and in the case of, for example, quartz crystal microbalance with dissipation monitoring (QCM-D)^58,59^ or multiparametric SPR,^60^ also film thickness. However, with these methods, it is difficult to precisely quantify the distance across which the biomolecular interactions between the nanoparticles and an SLB occur, i.e., the actual distance separating the bound nanoparticles from the SLB.

One alternative method that is particularly well suited to quantify biomolecular film thickness and coverage is neutron reflectometry (NR). In particular, aided by the possibility to vary the scattering length density (SLD) contrast^61,62^ between the substrate, the adsorbed film, and the background buffer by exchanging protium (^1^H) with deuterium (^2^H), NR has been shown to offer absolute thickness determinations with sub-nanometer precision of biomolecular films^63^ and their internal structure,^64^ even for multilayered systems.^65^ In the case of SLBs, NR was not only successfully used to quantify minute differences in the thickness of hydrophobic and hydrophilic portions of SLBs made from different lipid compositions^66^ but also structural alterations induced by changes in environmental conditions^67,68^ and biomolecular interactions.^69^ However, while thickness determination is relatively straightforward, separation distance determination is particularly challenging using NR, as NR requires strong SLD contrasts in the direction orthogonal to the interface and a well-defined layered structure, so far only realized for homogeneous flat layers.^12,70,71^ Thus, due to the geometry of spherically shaped nanoparticles, NR has been rarely used for in-depth characterization of adsorbed nanoparticles. In most of the cases reported in the literature, the investigations focused on the effect of NPs on the structure of the underlying layers, e.g., SLBs, and on the quantification of the adsorbed amount,^72,73^ without addressing the inherent possibility to characterize the structure, morphology, and separation distance between the substrate and the NP layer.

In this work, we demonstrate that by carefully designing the experiments and combining different theoretical models, NR can indeed be used to quantify the distance separating an SLB from naked and lipid-coated silica nanoparticles, as well as polydisperse hollow lipid vesicles. The distance between nanoparticles/vesicles and SLBs was varied by exploiting either direct nonspecific adsorption or specific binding. The latter was achieved via hybridization between cholesterol-modified DNA molecules, which were self-incorporated in the planar SLB and in the outer surface of lipid-coated NPs and hollow lipid vesicles. The DNA complexes were designed to adopt (i) a rod configuration^74^ to enforce a gap between the nanoparticles and the SLB or (ii) a zipper configuration, previously designed to mimic the SNARE–protein complex.^75^ We also show that NR provides an in-depth characterization that goes beyond the determination of separation distances. In fact, it provides precise information on the composition of adsorbed biological nanoparticles, their surface coverage and size distribution, as well as potential structural changes in the planar SLB upon interaction with nanoparticles or vesicles. The results refute a long-standing conviction in the field that NR cannot be used to study polydisperse biological nanoparticles. The unprecedented precision with which these parameters could be quantified should offer new means for an in-depth understanding of interactions between nanoparticles and interfaces occurring at nanometric separation distances, which is particularly relevant for investigations of the molecular recognition reactions of inorganic and biological nanoparticles with cellular membranes.

## Results and Discussion

**Figure 1:**
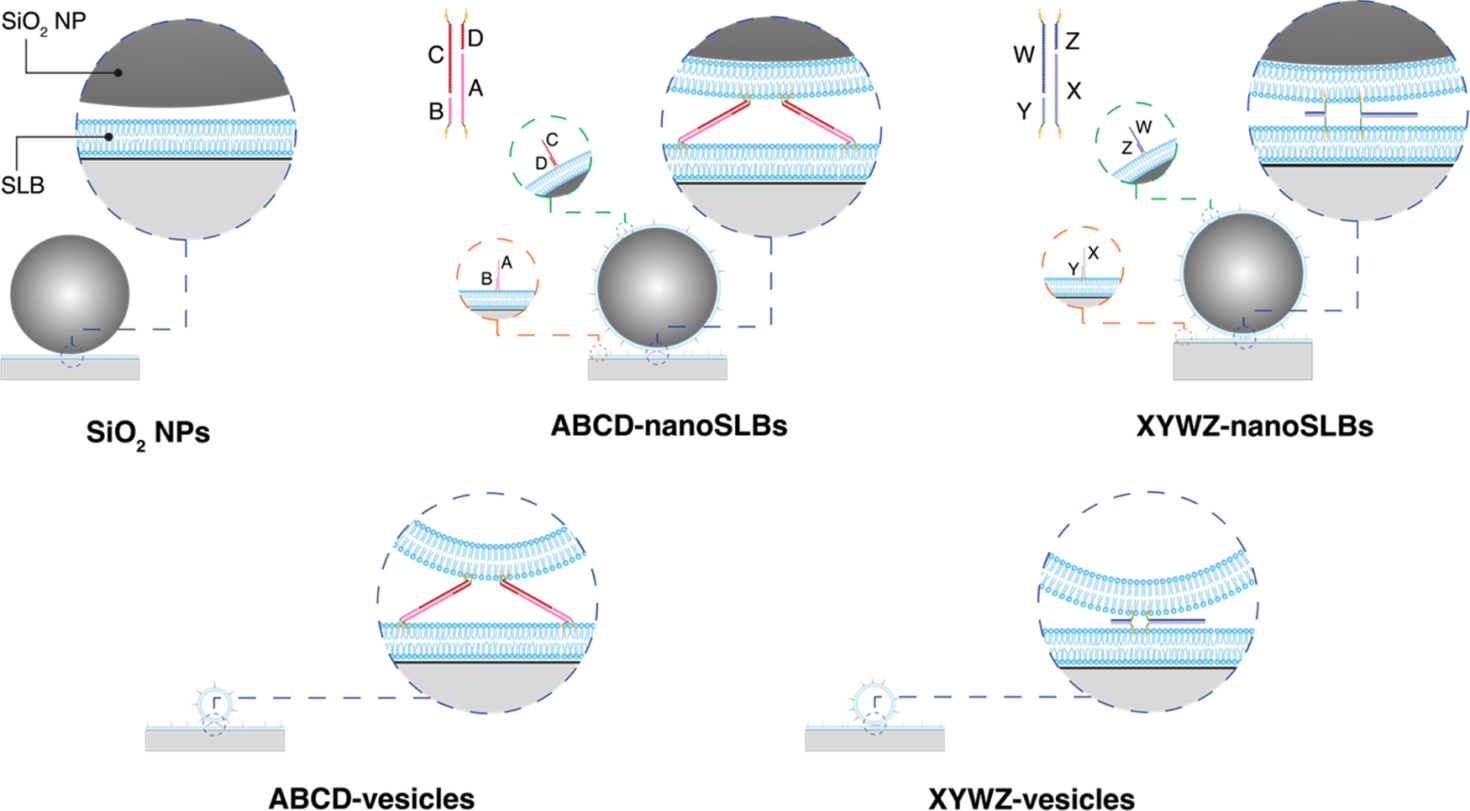
Schematics representing the five systems investigated in this work. (i) SiO_2_ NPs: directly adsorbed SiO_2_ NPs to a planar SLB; (ii) ABCD-nanoSLBs: lipid-coated SiO_2_ NPs (referred to as nanoSLBs) attached to a planar SLB via extended DNA (referred to as ABCD-DNA); (iii) XYWZ-nanoSLBs: nanoSLBs attached to a planar SLB via zipper DNA (referred to as XYWZ-DNA); (iv) ABCD-vesicles: lipid vesicles attached to a planar SLB via ABCD-DNA; (v) XYWZ-vesicles: lipid vesicles attached to a planar SLB via XYWZ-DNA. Though the schematics are approximately proportional to real values, they are not drawn to scale. Detailed step-by-step schematics describing the experimental procedure for each system are given in Figure S2.

To cover different separation distances between planar SLBs and adsorbed nanoparticles as well as different nanoparticle compositions and size distributions, the following five systems formed on planar SLBs were investigated (Figure 1):

(i) direct adsorption of SiO_2_ NPs;
(ii) binding of lipid-coated SiO_2_ NPs (referred to as nanoSLBs) via extended DNA (referred to as ABCD-DNA);
(iii) binding of nanoSLBs via zipper DNA (referred to as XYWZ-DNA);
(iv) binding of hollow lipid vesicles via ABCD-DNA;
(v) binding of hollow lipid vesicles via XYWZ-DNA.

A thorough characterization of the SiO_2_ NPs, nanoSLBs, and lipid vesicles was conducted before running the NR experiments. The sphericity of the SiO_2_ NPs was independently confirmed using scanning electron microscopy and atomic force microscopy (AFM).^40^ Their diameter was also independently determined and found to be 143 and 146 ± 2 nm according to AFM^40^ and dynamic light scattering (DLS; Figure S1) measurements, respectively. The vesicles had an average diameter, *Z*_avg_ = 66.8 ± 0.1 nm, and polydispersity index (PDI) of 0.06 ± 0.01 (Figure S1), as determined using DLS. Control experiments to ensure the proper formation of the nanoSLBs (Figure S7), optimize the experimental conditions (Figure S8), and rule out nonspecific binding between the nanoSLBs/vesicles and the planar SLB (Figures S9–S12), were conducted by means of the QCM-D technique.

The NR data were collected and analyzed systematically after each adsorption step to characterize (i) the bare silicon block, (ii) the planar SLBs formed on the surface of the silicon block, (iii) the DNA-decorated planar SLBs (for the relevant experiments), and (iv) the nanoparticle/vesicle layer. To achieve an unambiguous fitting of the data, we applied the contrast variation method,^61^ collecting NR data in aqueous buffers with various D_2_O/H_2_O mixing ratios, as explained hereafter. The measured neutron reflectivity depends on the so-called scattering length density (SLD), which is a volumetric average of the nuclear scattering length of the atoms that constitute a certain molecule. The difference between the SLD of different regions within the sample defines the contrast. SLD is also isotope-dependent and is, therefore, different for hydrogen-rich and deuterium-rich molecules. Indeed, SLDs for D_2_O and H_2_O are very different, namely, 6.35 × 10^–6^ Å^–2^ and −0.56 × 10^–6^ Å^–2^, respectively (at 25 °C). Thus, by varying the SLD of the aqueous buffers, the contrast of the adsorbed layers with respect to the background buffer was varied, allowing to reveal different structural features of the sample and to enhance the robustness of the data modeling. In addition to D_2_O and H_2_O, NR data were collected in D_2_O/H_2_O mixtures with nominal SLDs of 3.47 × 10^–6^ Å^–2^ and 2.07 × 10^–6^ Å^–2^. The former mixture was chosen to match the SLD of SiO_2_ (null contrast between the buffer and SiO_2_), here referred to as SiO_2_-matched water (SiO_2_MW), while the latter mixture was selected to match the SLD of Si (null contrast between the buffer and Si) and is therefore referred to here as Si-matched water (SiMW). A detailed account of the experiments and all solutions used are provided in the Materials and Methods section, together with schematics showing the step-by-step procedure for each experiment.

### Bare Silicon Blocks, SLBs, and DNA-Decorated SLBs

NR data and fits for the bare silicon blocks in D_2_O and H_2_O (Figure S13a) revealed SiO*_x_* layers with thicknesses, *t*_SiO*_x_*_ ≈ 1 nm, for all blocks, with a low but variable interfacial roughness, σ_SiO*_x_*_ = 3.5–5.5 Å (Table S1). The experimental NR data and the fits for the planar SLBs formed on top of the silicon blocks are shown in Figure S13b. The results demonstrate that the SLBs formed on all blocks were of high quality, with an interfacial roughness σ_SLB_ ≤ 5.2 Å and covering 96 to >99% of the solid surface exposed to the neutron beam (Table S1 and Figure S13c). For all SLBs, the modeling was performed using the lipid plugin of the NR fitting software, Aurore.^76^ The plugin applies several molecular constraints to ensure that the fitted parameters represent a physically realistic SLB. NR measurements were also performed on SLBs after *in situ* binding of AB-DNA or XY-DNA. In both cases, there were no detectable differences between the NR data before (i.e., that of pristine SLB layers) and after the DNA binding step. Overlayed NR spectra before and after the DNA binding step are shown in Figure S14. First, these results show that there is no detectable change in the SLB structure after adding the DNA molecules. Second, the bound DNA had a volume fraction below the detection limit of NR. Indeed, using NR, the anchoring of the DNA to the SLBs can only be indirectly confirmed via the binding of nanoSLBs and vesicles decorated with the complementary DNA strands. As confirmed using QCM-D, the absence of DNA on either the SLBs or nanoSLBs/vesicles resulted in no attachment of the nanoSLBs/vesicles (Figures S9–S12). Altogether, these results provide an accurate characterization of the high-quality SLBs formed, which is imperative for a successful and unambiguous interpretation of the NR data collected upon the subsequent formation of the nanoparticle/vesicle layers investigated in this work.

### SiO_2_ NPs

**Figure 2:**
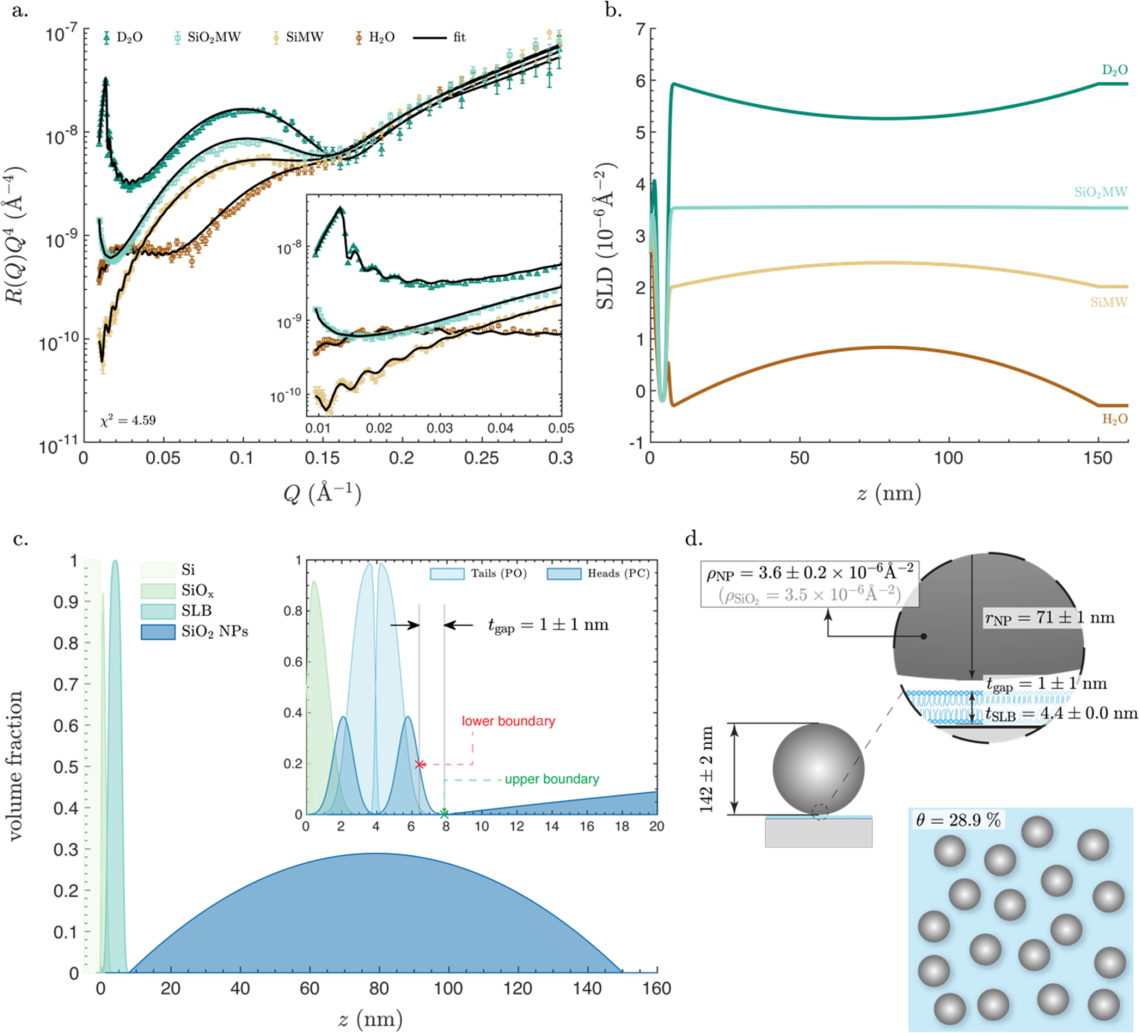
Characterization of SiO_2_ NP layer directly adsorbed to a planar SLB. (a) Neutron reflectometry data and fits in aqueous buffers with four different H_2_O and D_2_O mixing ratios: (i) D_2_O: nominal SLD 6.35 × 10^–6^ Å^–2^; (ii) SiO_2_MW: nominal SLD 3.47 × 10^–6^ Å^–2^; (iii) SiMW: nominal SLD 2.07 × 10^–6^ Å^–2^; (iv) H_2_O: nominal SLD −0.56 × 10^–6^ Å^–2^. The likelihood parameter χ^2^ is also indicated. The inset shows a zoomed-in section of the plot at the low-*Q* range. (b) SLD profiles versus the perpendicular distance from the surface of the silicon block, *z*, in the four different aqueous solutions. (c) Volume fraction profiles (VFPs) of all components of the system: the silicon block (Si), the SiO*_x_* layer on top, the SLB, and the SiO_2_ NP layer. The inset shows a zoomed-in section, depicting the VFPs of the heads and tails layers composing the planar SLB and the separation distance between the SLB and the SiO_2_ NP layer, *t*_gap_, which represents the distance between the inflection point of the VFP of the outer headgroups (indicated as the lower boundary with red x) and the first nonzero value for the VFP of the SiO_2_ NP layer (indicated as the upper boundary with green x). (d) Artistic schematics showing lateral and in-plane views of the system, with all of the fitted parameters noted on the schematic: thickness of the planar SLB, *t*_SLB_, radius of the SiO_2_ NPs, *r*_NP_, SLD of the NPs, ρ_NP_, separation distance, *t*_gap_, and surface coverage, θ. Though the schematics are approximately proportional to real values, they are not drawn to scale. All model parameters are listed in Table S2.

Figure 2a shows the reflectivity curves measured in D_2_O, SiO_2_MW, SiMW, and H_2_O, for the SiO_2_ NP layer adsorbed to a planar SLB. Particularly noticeable is that the reflectivity curves in D_2_O, SiMW, and H_2_O exhibited high-frequency fringes in the low-*Q* range (Figure 2a, inset), which is a characteristic feature of thick films, much thicker than the planar SLB. Conversely, the reflectivity curve in SiO_2_MW lacked such fringes. Indeed, the SLD matching between the SiO_2_MW and the SiO_2_ NPs makes the SiO_2_ NPs invisible to the neutron beam. Figure 2b shows the resulting SLD profiles for the four measurements versus *z*, i.e., the perpendicular distance from the surface of the silicon block. In accordance with the reflectivity data, the SiO_2_ NPs are not visible in the SLD profile obtained from the SiO_2_MW data. As expected, in the other three solutions, the magnitude of the peak-of-curvature in the same *z* range is proportional to the difference between the SLD of the SiO_2_ NPs and that of the solution. Although SLD profiles are informative for an experienced eye, volume fraction profiles (VFPs) provide more direct information about the location of different molecular species in the system. Figure 2c shows the VFPs for all of the system components, including the underlying SLB layer, SiO*_x_* layer, and the silicon block. The separation distance between the SLB and the SiO_2_ NPs is *t*_gap_ = 1 ± 1 nm, which is defined as the distance between the inflection point of VFP of the outer headgroups of the planar SLB and the first nonzero value for the VFP of SiO_2_ NP layer. Such a small separation distance is anticipated because the bare SiO_2_ NPs attach directly to the SLB, thus being only separated by very short-range forces. It is worth noting that, within the experimental accuracy, the separation distance between the planar SLB and SiO_2_ NPs, *t*_gap_ = 1 ± 1 nm is indistinguishable from the separation distance between the silicon blocks and planar SLBs, ∼0.5 ± 0.2 nm (Table S1).

The values of all of the model parameters obtained from the analysis of the NR curves are summarized in Figure 2d, revealing that the size of the SiO_2_ NPs is 2 × *r*_NP_ = 142 ± 2 nm, which is in excellent agreement with the size estimation obtained by AFM (143 nm)^40^ and DLS (146 ± 2 nm in Figure S1) on the same NP batch. The slightly larger value obtained by DLS is expected as the technique probes the hydrodynamic size of particles, which is often larger than their actual size.

It is also worth mentioning that the model used for the data fitting allowed for direct determination of *r*_NP_ and the water fraction in the SiO_2_ NP layer, *f*_w,NP_. This was achieved by directly including *f*_w,NP_ and the VFP of a homogeneous sphere defined numerically as a function of *r*_NP_ in the modeling (for details, see the Materials and Methods section). Adopting this approach avoided any ambiguity in defining the geometry of the NP layer, which would have otherwise arisen if a standard slab model were to be used. The fitted SLD value of the SiO_2_ NPs, ρ_NP_ = 3.6 ± 0.2 × 10^–6^ Å^–2^, is in full agreement with the theoretical value of SiO_2_, ρ_SiO_2__ = 3.5 × 10^–6^ Å^–2^. Finally, based on *f*_w,NP_, we calculated the surface coverage of the SiO_2_ NPs to be θ = 28.9%, which is in the expected range below the jamming limit of ∼55% for monodispersed spheres randomly adsorbing to a surface.^77^ A surface coverage lower than the jamming limit is likely due to adsorption of the NPs under stagnant, i.e., no flow, conditions. Under such conditions, one can expect local depletion of the NP concentration near the SLB interface, which may attenuate the final surface coverage.

NR also offers the unique opportunity to probe the NP layer while simultaneously monitoring changes in the underlying SLB. This is possible because the distance probed by NR is, to a good approximation, inversely proportional to *Q* and equals . Therefore, the NR signal of layers with thickness ≫ 15 nm, such as the SiO_2_ NP layer, will primarily result in fringes in reflectivity in the low-*Q* range (*Q* < 0.04 Å^–1^), and thinner layers, such as the SLB, will mainly contribute to the overall shape of the reflectivity curve, with features appearing, if any, in the mid and high-*Q* range (*Q* > 0.05 Å^–1^). In fact, no detectable changes in the overall shape of the reflectivity curves were observed before and after the adsorption of the NPs (Figure S15), indicating that despite the formation of an NP layer in close proximity to the SLB, the NPs did not penetrate the SLB layer nor was there any indication of wrapping of lipids around the NPs.

### ABCD-nanoSLBs

**Figure 3:**
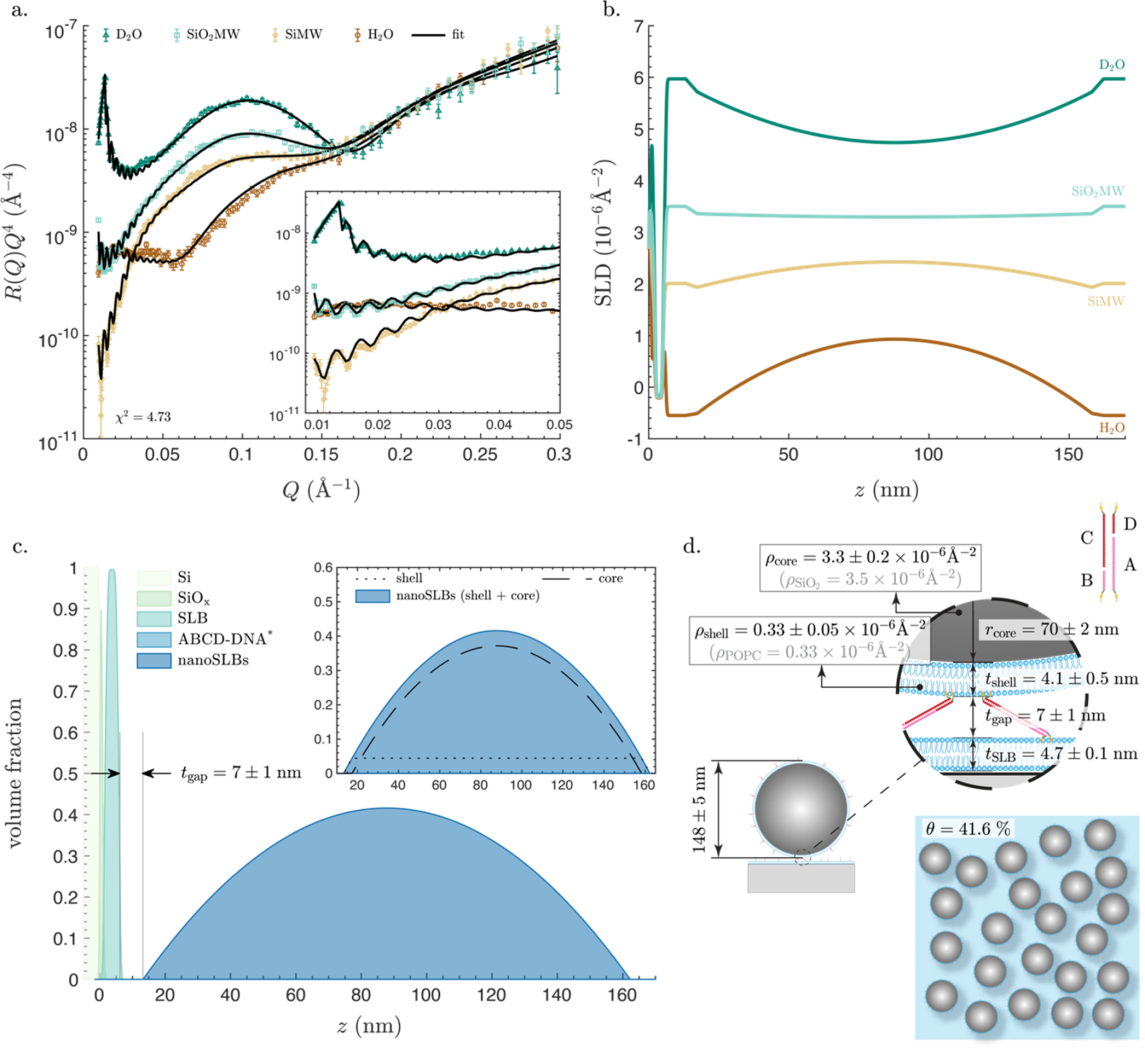
Characterization of a nanoSLB layer attached to a planar SLB using ABCD-DNA. (a) Neutron reflectometry data and fits in aqueous buffers with four different H_2_O and D_2_O mixing ratios: (i) D_2_O: nominal SLD 6.35 × 10^–6^ Å^–2^; (ii) SiO_2_MW: nominal SLD 3.47 × 10^–6^ Å^–2^; (iii) SiMW: nominal SLD 2.07 × 10^–6^ Å^–2^; (iv) H_2_O: nominal SLD −0.56 × 10^–6^ Å^–2^. The likelihood parameter χ^2^ is also indicated. The inset shows a zoomed-in section of the plot at the low-*Q* range. (b) SLD profiles versus the perpendicular distance from the surface of the silicon block, *z*, in the four different aqueous solutions. (c) Volume fraction profiles (VFPs) of all components of the system: the silicon block (Si), the SiO*_x_* layer on top, the SLB, the ABCD-DNA layer, and the nanoSLB layer. The separation distance, *t*_gap_, is also noted, which represents the distance between the inflection point of the VFP of the outer headgroups of the planar SLB and the first nonzero value for the VFP of the nanoSLB layer. The inset shows a zoomed-in section, depicting the VFPs of the SiO_2_ cores, the lipid shells, and the sum of both, which represents the VFP of the nanoSLBs. *The ABCD-DNA molecules had a negligible volume fraction. (d) Artistic schematics showing lateral and in-plane views of the system, with all of the fitted parameters noted on the schematic: thickness of the planar SLB, *t*_SLB_, radius and SLD of the core, *r*_core_ and ρ_core_, thickness and SLD of the shell, *t*_shell_ and ρ_shell_, separation distance, *t*_gap_, and surface coverage, θ. Though the schematics are approximately proportional to real values, they are not drawn to scale. All model parameters are listed in Table S3.

Unlike the SiO_2_ NPs, which are single-component particles, nanoSLBs are two-component particles: a lipid shell and a silica core. To account for both, the VFP used to fit the NR data was built by superimposing the individual VFPs of a homogeneous sphere and a spherical shell. The resulting nanoSLB VFP was described by five fitting parameters: the radius and the SLD of the SiO_2_ core, *r*_core_ and ρ_core_, the thickness and the SLD of the lipid shell, *t*_shell_ and ρ_shell_, and the solution volume fraction, *f*_w,nanoSLB_. Using this approach, excellent fits to the experimental data were achieved (Figure 3a). One might at first thought assume that a thin lipid layer, <5 nm, coating a much larger NP, ∼150 nm, would have an insignificant contribution to the NR signal; however, the reflectivity curves (Figure 3a) and the corresponding SLD profiles (Figure 3b) show otherwise. Unlike the SiO_2_ NPs, the reflectivity of the nanoSLB layer in SiO_2_MW exhibited fringes in the low-*Q* range (Figure 3a, inset), demonstrating that the presence of the lipid shell renders the nanoSLBs visible even if the SiO_2_ core is contrast-matched (Figure 3b). If one were to ignore the multicomponent structure of the nanoSLBs and try to model them as a one-component entity, no acceptable fitting of the data would be achievable (Figure S16). This ability to distinguish the different components of biological nanoparticles, e.g., the core and the shell, may be paramount to understanding biological processes occurring at the cell membrane interface, for example, the release of a viral genome from the capsid during membrane fusion.

The VFPs of the nanoSLB layer and the underlying layers are shown in Figure 3c, revealing *t*_gap_ = 7 ± 1 nm. The value is, as expected, larger than *t*_gap_ of the SiO_2_ NPs (1 ± 1 nm; Figure 2c, inset) and smaller than the theoretical length of completely extended 45 DNA base pairs of ∼15 nm. The separation distance also suggests that the DNA linkers are either at a nonperpendicular angle to the surface or have a secondary winded structure. As indicated by the small error of 1 nm, the quality of the fit is very sensitive to any variations in *t*_gap_; trying to fit the data while forcing *t*_gap_ outside the optimal range results in fits with substantially lower quality (Figure S17). Figure 3d summarizes the results of the fitted parameters, showing the anticipated size and composition of both the core and the shell: 2 × *r*_core_ = 140 ± 4 nm (within the error ranges indistinguishable from the SiO_2_ NP size determined using AFM, DLS, and NR in Figure 2d ), ρ_core_ = 3.3 ± 0.2 × 10^–6^ Å^–2^ (in perfect agreement with ρ_SiO_2__ = 3.5 × 10^–6^ Å^–2^), *t*_shell_ = 4.1 ± 0.5 nm (overlapping the range of values reported for planar SLBs, i.e., between 4.3 ± 0.1 nm and 4.7 ± 0.1 nm, Table S1), and ρ_shell_ = 0.33 ± 0.05 × 10^–6^ Å^–2^ (identical to the SLD of the POPC lipids composing the shell, ρ_POPC_ = 0.33 × 10^–6^ Å^–2^). The nanoSLBs had a surface coverage of θ = 41.6%, also within the expected range. Similar to the SiO_2_ NPs, the results show no sign of change in the structure of the underlying planar SLB (Figure S18); similarly, the results for XYWZ-nanoSLBs (Figure S19), ABCD-vesicles (Figure S20), and XYWZ-vesicles (Figure S21) exhibit no signs of structural changes of the underlying planar SLB.

### XYWZ-nanoSLBs

**Figure 4:**
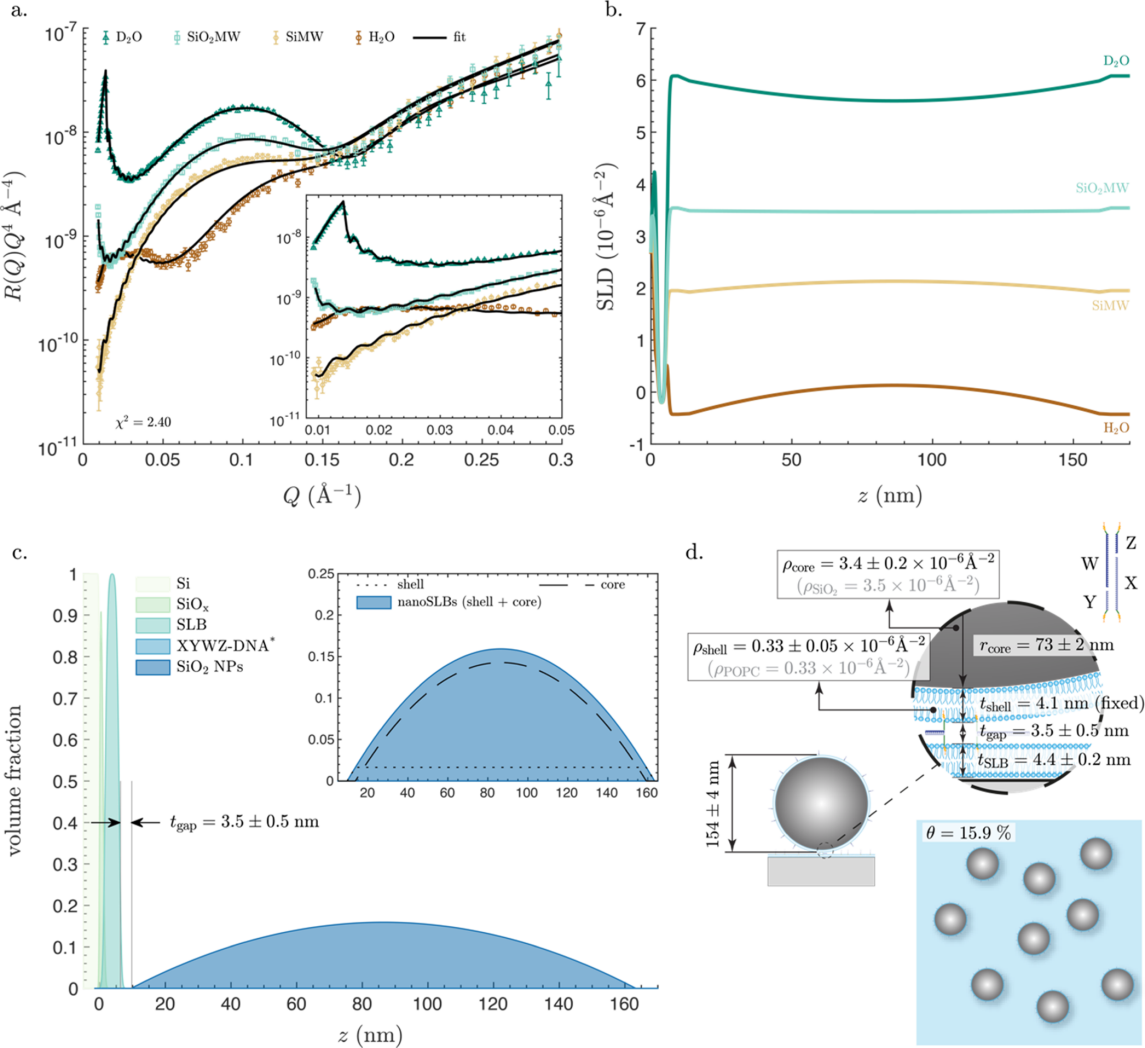
Characterization of a nanoSLB layer attached to a planar SLB using XYWZ-DNA. (a) Neutron reflectometry data and fits in aqueous buffers with four different H_2_O and D_2_O mixing ratios: (i) D_2_O: nominal SLD 6.35 × 10^–6^ Å^–2^; (ii) SiO_2_MW: nominal SLD 3.47 × 10^–6^ Å^–2^; (iii) SiMW: nominal SLD 2.07 × 10^–6^ Å^–2^; (iv) H_2_O: nominal SLD −0.56 × 10^–6^ Å^–2^. The likelihood parameter χ^2^ is also indicated. The inset shows a zoomed-in section of the plot at the low-*Q* range. (b) SLD profiles versus the perpendicular distance from the surface of the silicon block, *z*, in the four different aqueous solutions. (c) Volume fraction profiles (VFPs) of all components of the system: the silicon block (Si), the SiO*_x_* layer on top, the SLB, the XYWZ-DNA layer, and the nanoSLB layer. The separation distance, *t*_gap_, is also noted, which represents the distance between the inflection point of the VFP of the outer headgroups of the planar SLB and the first nonzero value for the VFP of the nanoSLB layer. The inset shows a zoomed-in section, depicting the VFPs of the SiO_2_ cores, the lipid shells, and the sum of both, which represents the VFP of the nanoSLBs. *The XYWZ-DNA molecules had a negligible volume fraction. (d) Artistic schematics showing lateral and in-plane views of the system, with all of the fitted parameters noted on the schematic: thickness of the planar SLB, *t*_SLB_, radius and SLD of the core, *r*_core_ and ρ_core_, thickness and SLD of the shell, *t*_shell_ and ρ_shell_, separation distance, *t*_gap_, and surface coverage, θ. Though the schematics are approximately proportional to real values, they are not drawn to scale. All model parameters are listed in Table S4.

Figure 4a shows the reflectivity curves for the XYWZ-nanoSLBs. Similar to the ABCD-nanoSLBs, the curves exhibit high-frequency fringes in the low-*Q* range (Figure 4a, inset), indicative of the formation of a thick layer, much thicker than the planar SLB. However, the amplitude of the fringes in all aqueous solutions is damped compared to the ABCD-nanoSLBs, indicating substantially lower surface coverage. Under the same experimental conditions, it is expected that XYWZ-nanoSLBs will have lower surface coverage than ABCD-nanoSLBs due to differences in the configuration between the two DNA linkers. While ABCD-DNA is anchored with two connected cholesterol molecules per DNA linker at both the planar SLB and the nanoSLB ends, the XYWZ-DNA is anchored with only one cholesterol molecule per DNA linker at both ends, due to the separation into two linkers upon hybridization of the XY- with WZ-DNA, forming an XW linker and a YZ linker (Figure 1).^75^ It has been previously shown that DNA molecules attached with single cholesterol anchors bind reversibly to lipid bilayers, while those attached with double anchors bind essentially irreversibly.^78,79^ This difference is likely due to the continuous switching of cholesterol molecules between the aqueous and lipid phases. Such configuration allows only XYWZ-nanoSLBs with a sufficiently large number of linkers to remain attached to the planar SLB as illustrated in Figure S22.

At such low surface coverage, it is more challenging to resolve details such as the thickness of the lipid shell, as the layer contrast is directly proportional to the adsorbed amount. For data fitting, we have thus used the same approach as for the ABCD-nanoSLBs, but with fixing the shell thickness to the same value obtained from fitting the ABCD-nanoSLBs, *t*_shell_ = 4.1 nm. The resulting SLD profiles are very similar to that of the ABCD-nanoSLBs, but with attenuated curvature at the *z* range where the nanoSLBs are located (Figure 4b), confirming the lower surface coverage of the XYWZ-nanoSLBs. Still, the corresponding VFPs reveal the key difference between the two nanoSLB systems, namely, *t*_gap_ = 3.5 ± 0.5 nm (Figure 4c), which is half that of the ABCD-nanoSLBs. Indeed, the determined separation distance lies precisely in the expected range: larger than that of the SiO_2_ NPs (1 ± 1 nm) and smaller than ABCD-nanoSLBs (7 ±1 nm). All other fitting parameters, i.e., *r*_core_, ρ_core_, and ρ_shell_ (Figure 4d), were in perfect agreement with both the expected values and the ones determined for the ABCD-nanoSLBs. As indicated from the reflectivity curves, SLD profiles, and VFPs, the surface coverage of XYWZ-nanoSLBs was substantially low at θ = 15.9%.

### ABCD-Vesicles

The results from the SiO_2_ NPs, ABCD-nanoSLBs, and XYWZ-nanoSLBs demonstrated the successful use of NR to resolve the separation distances between planar SLBs and silica-based nanoparticles with sub-nanometer accuracy while revealing insightful details about their structure and composition. Many biological NPs are, however, more polydisperse and less dense than the so far investigated systems. Encouraged by the results of the SiO_2_ NPs and nanoSLBs experiments presented above, we decided to investigate the use of NR to resolve the separation distances of polydisperse, hollow lipids vesicles. In fact, with their hollow structure and relatively broad size distribution (Figure S1), the lipid vesicles used here are likely to be more challenging to obtain a sufficient signal than from most native biological NPs samples. This is so because most biological NPs, such as purified virus samples have a relatively tight size distribution,^80^ and their cores are at least partially filled with the viral genome, which makes them likely to be more visible to NR and easier to analyze than polydisperse and hollow lipid vesicles. This system thus represents a challenging experimental scenario, serving as a benchmark to define the limits of the approach.

**Figure 5:**
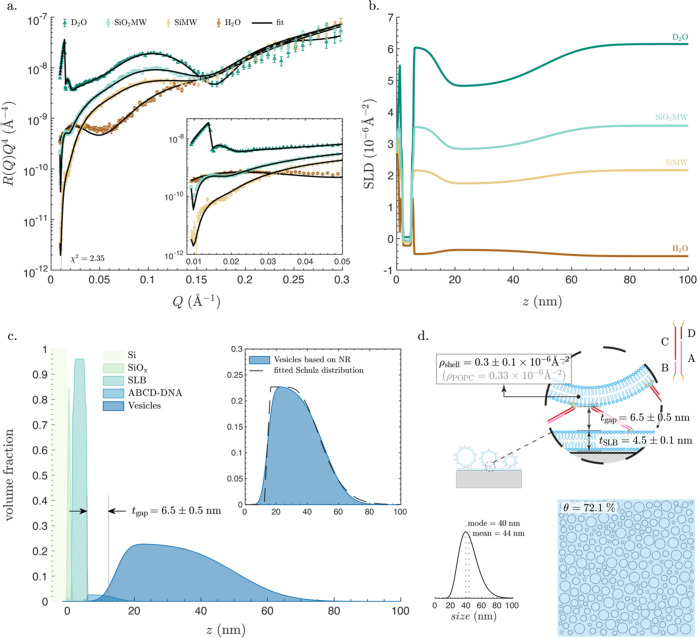
Characterization of a vesicle layer attached to a planar SLB using ABCD-DNA. (a) Neutron reflectometry data and fits in aqueous buffers with four different H_2_O and D_2_O mixing ratios: (i) D_2_O: nominal SLD 6.35 × 10^–6^ Å^–2^; (ii) SiO_2_MW: nominal SLD 3.47 × 10^–6^ Å^–2^; (iii) SiMW: nominal SLD 2.07 × 10^–6^ Å^–2^; (iv) H_2_O: nominal SLD −0.56 × 10^–6^ Å^–2^. The likelihood parameter χ^2^ is also indicated. The inset shows a zoomed-in section of the plot at the low-*Q* range. (b) SLD profiles versus the perpendicular distance from the surface of the silicon block, *z*, in the four different aqueous solutions. (c) Volume fraction profiles (VFPs) of all components of the system: the silicon block (Si), the SiO*_x_* layer on top, the SLB, the ABCD-DNA layer, and the vesicle layer. The separation distance, *t*_gap_, is also noted, which represents the distance between the inflection points of the VFP of the outer headgroups of the planar SLB and that of vesicle layer minus *t*_shell_/2 (eq S15). The inset shows a zoomed-in section of the empirically determined VFP of the vesicles and an overlayed VFP based on the fitted Schutz distribution. (d) Size distribution of the vesicles and an artistic schematic showing lateral and in-plane views of the system, with all of the fitted parameters noted on the schematic: thickness of the planar SLB, *t*_SLB_, SLD of the shell, ρ_shell_, separation distance, *t*_gap_, and surface coverage, θ. Though the schematics are approximately proportional to real values, they are not drawn to scale. All model parameters are listed in Table S5.

Figure 5a shows the reflectivity curves obtained for the ABCD-vesicle layer. Reflectivity curves in D_2_O, SiO_2_MW, and SiMW showed fringes in the low-*Q* range (Figure 5a, inset), but with a much lower frequency and amplitude compared to the SiO_2_ NP and the nanoSLB layers, indicative of the formation of a thinner layer. This result is expected given the smaller size of the vesicles (Figure S1). These fringes were almost invisible in the reflectivity curve collected in H_2_O, which was expected due to the reduced contrast (difference in SLD) between H_2_O and the POPC lipids composing the vesicles. In theory, one can use the same VFP-based fitting approach as was used for the SiO_2_ NPs and nanoSLBs to fit the NR data collected upon the formation of a vesicle layer. However, this would require accounting for the size distribution when calculating *r*_core_. Unfortunately, such an approach comes at a high computational cost, hindering successful data fitting within reasonable time frames. Therefore, the NR data were analyzed using a modified version of the standard slab model in which the vesicle layer was described by a slab with asymmetric roughness on both sides. The resulting SLD profiles (Figure 5b) reveal a small roughness at the interface close to the planar SLB and a much larger roughness at the interface between the vesicles and the bulk water solution, indicative of the arrangement and broad size distribution of the attached vesicles. The resulting VFPs (Figure 5c) reveal a separation distance, *t*_gap_ = 6.5 ± 0.5 nm, which within the error ranges is indistinguishable from the separation distance of the ABCD-nanoSLB layers.

We then used the empirically determined VFP of the vesicle layer (extracted from the SLD profiles) to determine the size distribution of the adsorbed vesicles (Figure 5c, inset). This calculation was performed assuming spherical vesicles with a shell thickness of 4.1 nm (equivalent to the shell thickness of the nanoSLBs) and size distribution described by the Shultz function;^81^ detailed calculations are provided in the Materials and Methods section. The size distribution of the adsorbed vesicles shifted toward smaller sizes (Figure 5d) with respect to that obtained by DLS for the same batch of vesicles in solution (Figure S1). This observation is in line with the well-documented bias for adsorption of smaller entities in a pool of polydisperse particles, a phenomenon generally attributed to the faster diffusion of smaller objects.^82^ Moreover, in the case of random sequential adsorption, as adsorption progresses over time, the remaining spaces between the adsorbed vesicles will accommodate only smaller vesicles, which could also contribute to the observed shift in size distribution toward smaller sizes of the attached vesicles. As described in the Materials and Methods section and depicted in Figure S6, using this size distribution, we could accurately determine the surface coverage to be θ = 72.1%.

### XYWZ-Vesicles

**Figure 6:**
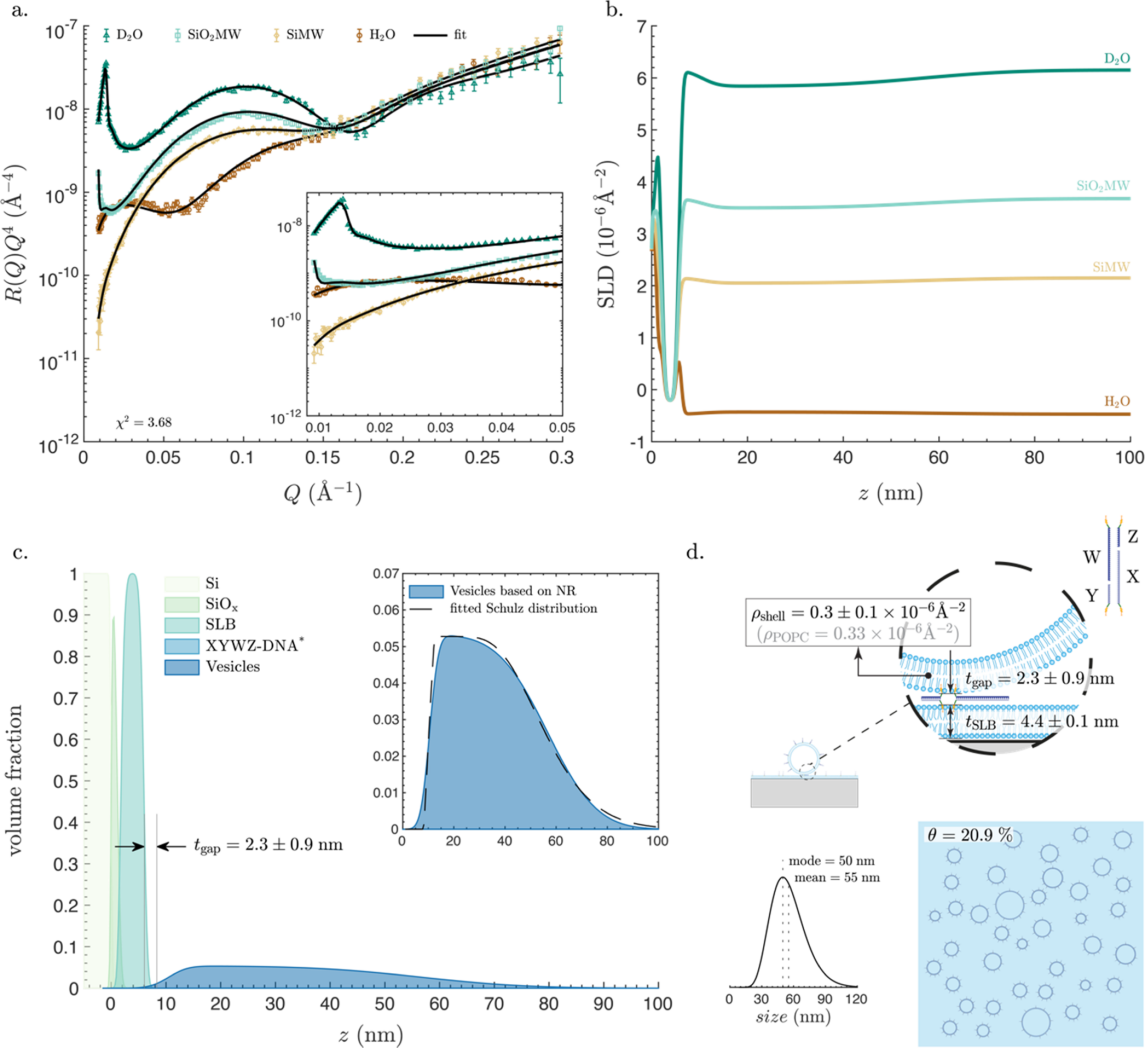
Characterization of a vesicle layer attached to a planar SLB using XYWZ-DNA. (a) Neutron reflectometry data and fits in aqueous buffers with four different H_2_O and D_2_O mixing ratios: (i) D_2_O: nominal SLD 6.35 × 10^–6^ Å^–2^; (ii) SiO_2_MW: nominal SLD 3.47 × 10^–6^ Å^–2^; (iii) SiMW: nominal SLD 2.07 × 10^–6^ Å^–2^; (iv) H_2_O: nominal SLD −0.56 × 10^–6^ Å^–2^. The likelihood parameter χ^2^ is also indicated. The inset shows a zoomed-in section of the plot at the low-*Q* range. (b) SLD profiles versus the perpendicular distance from the surface of the silicon block, *z*, in the four different aqueous solutions. (c) Volume fraction profiles (VFPs) of all components of the system: the silicon block (Si), the SiO*_x_* layer on top, the SLB, the XYWZ-DNA layer, and the vesicle layer. The separation distance, *t*_gap_, is also noted, which represents the distance between the inflection points of the VFP of the outer headgroups of the planar SLB and that of vesicle layer minus *t*_shell_/2 (eq S15). The inset shows a zoomed-in section of the empirically determined VFP of the vesicles and an overlayed VFP based on the fitted Schutz distribution. *The XYWZ-DNA molecules had a negligible volume fraction. (d) Size distribution of the vesicles and an artistic schematic showing lateral and in-plane views of the system, with all of the fitted parameters noted on the schematic: thickness of the planar SLB, *t*_SLB_, SLD of the shell, ρ_shell_, separation distance, *t*_gap_, and surface coverage, θ. Though the schematics are approximately proportional to real values, they are not drawn to scale. All model parameters are listed in Table S6.

The reflectivity curves for the XYWZ-vesicle layer (Figure 6a) showed largely attenuated features compared to the ABCD-vesicle layer. Indeed, features originating from the presence of the vesicles were only visible in D_2_O and SiO_2_MW, the aqueous solutions with the largest SLD difference from the POPC lipids. However, it was still possible to fit the data using the same approach used for the ABCD-vesicles. The partial absence of features in NR data of the vesicle layer was reflected in all four SLD profiles (Figure 6b), featuring a relatively flat, smooth profile in the *z* region where the vesicles are located. All of these observations indicate substantially lower surface coverage for the XYWZ-vesicles than the ABCD-vesicles; the lower surface coverage is expected due to the same reasons discussed for the XYWZ-naonSLBs and illustrated in Figure S22, allowing only vesicles with a sufficient number of DNA linkers to remain attached. The VFPs (Figure 6c) reveal that the volume fraction of the vesicles peaked at ∼5%, a remarkably low value. The corresponding separation distance between the vesicle and the planar SLB was determined to be *t*_gap_ = 2.3 ± 0.9 nm, which within the error ranges is indistinguishable from the distance determined for XYWZ-nanoSLBs, *t*_gap_ = 3.5 ± 0.5 nm. The size distribution determined by fitting the VFP of the vesicle layer to the Schultz distribution function (Figure 6c, inset, and d) reveals a shift to larger vesicle sizes compared to that of the ABCD-vesicles. This shift is attributed to the higher number of DNA linkers needed for the XYWZ-vesicles to remain attached to the surface, which is preferentially accommodated by larger vesicles (Figure S22). Finally, the computed surface coverage (Figure S6) was found to be θ = 20.9%. While being even higher than the surface coverage of the XYWZ-nanosSLBs (θ = 15.9%), the volume fraction, which is the main parameter contributing to the NR signal, occupied by the vesicles is less than one-third that of the nanoSLBs, due to the hollow structure of the vesicles.

## Conclusions

In this work, we have shown that NR can be used to interrogate the interactions of biological nanoparticles with planar mimetic cell membranes, offering an integrated structural characterization. Most notable is the ability to measure the separation distance between the nanoparticles and the membranes with sub-nanometric accuracy, even for polydisperse samples at volume fraction coverages as low as 5%. With this precision, it is tempting to speculate about the potential possibility of using NR to, for example, investigate the calcium-facilitated structural change of the SNARE complex that brings synaptic vesicles toward the cellular membrane to initiate fusion with neuronal cells.^83,84^ Similarly, viruses have evolved equally fine-tuned spatiotemporal mechanisms to control cellular uptake and subsequent association and fusion with endosomal membranes to release their genetic cargo into the cytosol,^85,86^ a process that could also be addressed using NR.

The richness of the NR data also allowed us to determine the size and material composition of each component of the nanoparticles, the size distribution of the polydisperse samples, surface coverage, and even probe the possible structural changes in the underlying mimetic cell membrane. The possibility to characterize interactions, material composition, and structural changes within nanometer distances to cellular membranes is crucial for optimizing supramolecular assembly strategies for many biomedical applications, such as the intermolecular interactions at cellular membranes that inspired the design of the pH-sensitive lipid nanoparticles (LNPs) used for drug delivery, which was recently applied for mRNA vaccines that helped mitigate the COVID-19 pandemic.^87^ Additionally, applications for biosensor design and optimization, as well as interactions of nanoparticles at environmental interfaces, are also of direct relevance. Thus, given the unprecedented precision with which we quantified these crucial parameters, NR could offer an in-depth understanding of the interaction between nanoparticles and interfaces in general, and biological nanoparticles and cellular membranes in particular.

## Supporting Information Available

Detailed materials and methods with explanatory figures; tables summarizing the values of all of the modeling parameters; figures for results from DLS experiments to characterize the SiO_2_ NPs, nanoSLBs, and vesicles; preliminary experiments conducted using QCM-D; NR results for the silicon blocks and planar SLBs; overlayed before and after NR data; additional fitting results showing the sensitivity of the results to minor variations in the fitted parameters; and schematics showing the difference in binding configuration between the ABCD- and XYWZ-DNA (PDF)
