# Supplementary material for: Probing the Separation Distance between Biological Nanoparticles and Cell Membrane Mimics Using Neutron Reflectometry with Sub-Nanometer Accuracy
Source: J Am Chem Soc. 2022 Nov 3;144(45):20726–38. doi: 10.1021/jacs.2c08456 (PMC9673153; doi:10.1021/jacs.2c08456)
Supplement: Supplementary file 1 — ja2c08456_si_001.pdf [file ja2c08456_si_001.pdf]

Supporting Information for Probing the Separation Distance between Biological Nanoparticles and Cell Membrane Mimics Using Neutron Reflectometry with Sub-Nanometer Accuracy

Antonius Armanious*†[1], Yuri Gerelli*[2,3], Samantha Micciulla[2], Hudson P. Pace[1], Rebecca J.L. Welbourn[4], Mattias Sjöberg[1], Björn Agnarsson[1], and Fredrik Höök*[1]

[1]Department of Physics, Chalmers University of Technology, 41296 Gothenburg, Sweden
[2]Institut Max von Laue-Paul Langevin (ILL), 38042 Grenoble, France
[3]Department of Life and Environmental Sciences, Università Politecnica delle Marche, 60131 Ancona, Italy
[4]ISIS Facility, STFC, Rutherford Appleton Laboratory, Chilton, Didcot, Oxon OX11 0QX, United Kingdom

*armanioa@ethz.ch; y.gerelli@staff.univpm.it; fredrik.hook@chalmers.se
†Present address: Department of Health Sciences and Technology, ETH Zurich, Zurich, Switzerland

Materials and Methods

Chemicals and Materials

Sodium dodecyl sulfate (SDS; ≥ 99.0%), D_2O (99.9 atom % D), HCl (1 M), NaCl (≥ 99%), bis(2-hydroxyethyl) amino-tris-(hydroxymethyl)-methane (Bis-Tris; ≥ 98%), 150 nm SiO_2 nanoparticles (NPs), anhydrous chloroform (≥ 99%), and 1-palmitoyl-2-oleoyl-glycero-3-phosphocholine (POPC) were purchased from Merck Sigma-Aldrich (Darmstadt, Germany). Silicon single crystal blocks were purchased from Sil'tronix Silicon Technologies (Archamps, France). The following cholesteryl-TEG modified DNA oligonucleotides were purchased from Eurogentec (Seraing, Belgium).

- 5'-TGG-ACA-TCA-GAA-ATA-AGG-CAC-GAC-GGA-CCC-3'-TEG-Cholesterol (ssA)
- Cholesterol-TEG-5'-CCC-TCC-GTC-GTG-CCT-3' (ssB)
- 5'-TAT-TTC-TGA-TGT-CCA-AGC-CAC-GAG-TTC-CCC-3'-TEG-Cholesterol (ssC)
- Cholesterol-TEG-5'-CCC-GAA-CTC-GTG-GCT-3' (ssD)
- 5'-TGG-ACA-TCA-GAA-ATA-AGG-CAC-GAC-GGA-3'-TEG-Cholesterol (ssX)
- Cholesterol-TEG-5'-TCC-GTC-GTG-CCT-3' (ssY)
- Cholesterol-TEG-5'-TCC-GTC-GTG-CCT-TAT-TTC-TGA-TGT-CCA-3' (ssW)
- 5'-AGG-CAC-GAC-GGA-3'-TEG-Cholesterol (ssZ)

Buffers

All experiments were conducted in a buffer with pH (pD) = 7.0, 150 mM NaCl, and 10 mM Bis-Tris. Buffers were prepared both in H_2O (Milli-Q purity with resistivity ~ 18.2 Ω·cm; Merck Millipore, Molsheim, France) or D_2O (99.9 atom % D). For both buffers pH (pD) was adjusted using HCl 1M; pH (pD) was determined using a pH meter and pH paper for H_2O- and D_2O-based buffers, respectively. The H_2O-based buffer was sterilized by autoclaving at 120 ℃ for 20 minutes and stored in the dark until used. The D_2O-based buffer was freshly prepared directly before the start of the neutron reflectometry (NR) experiments.

POPC vesicles preparation

POPC dissolved in chloroform with concentration of $10\ \mathrm{mg \cdot ml^{-1}}$ was used to prepare the vesicles. 1 ml of the POPC solution was dried in a 50 ml round flask under vacuum at 25 ℃ using a rotavap setup and left under vacuum overnight to get rid of any residual chloroform. The dried POPC lipids were then hydrated using the H_2O-based buffer to a concentration of $1\ \mathrm{mg \cdot ml^{-1}}$. After ~ 30 min of incubation in buffer, the flask was briefly placed in a bath sonicator to dissolve any traces of lipids off the walls of the flask. The POPC solution was then subjected to five cycles of freeze/thawing using liquid N_2 and a 40 ℃ water bath. The remaining steps of the protocol varied based on the planned use of the vesicles. Two types of vesicles were prepared: one was prepared by tip-sonication followed by extrusion (referred to as 'sonicated-POPC' in the following), and the other was prepared by extrusion only (referred to as 'extruded-POPC' in the following).

Sonicated-POPC vesicles were prepared using a Vibra-Cell™Ultrasonic Liquid Processors from Sonics (USA). The sonication was applied using a tip immersed in the POPC solution at 30% of the maximum power with consecutive cycles of 2 s sonication and 3 s pause for a total duration of ~ 30 min. The tube containing the POPC solution was placed in ice to avoid overheating. Afterward, the solution was centrifuged at 20000 g to get rid of any debris from the tip of the sonicator. Finally, the supernatant POPC solution was extruded 11 times through a 30 nm polycarbonate filter (Whatman, UK) using a mini-extruder from Avanti (USA). The sonicated-POPC vesicles were used for the preparation of supported lipids bilayers both on flat Si block (SLBs) and on SiO_2 NPs (nanoSLBs).

The extruded-POPC vesicles were prepared by extruding the POPC solution 31 times through a 30 nm polycarbonate filter (Whatman, UK) using a mini-extruder from Avanti (USA). The extruded-POPC vesicles

were used to prepare the DNA-decorated vesicles for subsequent attachment to the DNA-decorated SLBs. The sonicated-POPC vesicles had a smaller but broader size distribution, and the extruded-POPC vesicles had a larger but narrower size distribution (Figure S1) as determined by dynamic light scattering (DLS) measurements.

nanoSLBs preparation

15 μl of 150 nm SiO_2 NPs (5%wt.) were added per 1 ml of sonicated-POPC (1 $mg \cdot mL^{-1}$); the corresponding surface area of the POPC bilayers in the vesicles is ~ 10 times higher than the surface area of SiO_2 NPs. The mixture was shortly vortexed and incubated for ~ 1 h at room temperature, followed by ~ 7 h at 4 ℃. Incubation for at least ~ 6 h is necessary for the successful formation of the nanoSLBs, i.e., the formation of the SLBs on the top of the SiO_2 NPs. Afterwards the nanoSLBs were transferred in 1.5 ml tubes and centrifuged at 20000 g for 10 min. The supernatant, ~ 1.45 ml, was decanted and the nanoSLBs palette was re-suspended in buffer by aggressive pipetting followed by vortexing for at least 10 s at 3000 rpm. The centrifuging and re-suspension steps were then repeated two more times to get rid of most of the residual vesicles. The successful formation of the nanoSLBs was verified by running DLS (Figure S1) and quartz crystal microbalance with dissipation monitoring (QCM-D) measurements (Figure S7).

DNA tethers preparation

5 μM AB-DNA, CD-DNA, XY-DNA, and WZ-DNA were formed by mixing equal volumes of 10 μM of the respective single-stranded DNA molecules; the solutions were vigorously pipetted and vortexed to ensure proper mixing. The solutions were then stored at 4 ℃ until use.

CD-nanoSLBs and WZ-nanoSLBs were prepared by mixing 7.2 μl CD-DNA or WZ-DNA (5 μM) per 1 ml of nanoSLBs (~ 2.2×10^{11} $particle \cdot ml^{-1}$), achieving a final density of ~ 100 DNA molecule per nanoSLB (≡ 1 DNA molecule per 707 nm^2). This is much lower than the saturation capacity of 315 DNA molecules per nanoSLB, estimated based on work by Pfeiffer and Höök (2006) [1]; therefore, we expect to have no freely floating DNA molecules in the nanoSLB solutions.

CD-Vesicles and WZ-Vesicles were prepared in a similar manner to that of the nanoSLBs targeting the same DNA density of ≡ 1 DNA molecule per 707 nm^2. For this end, 130 μl CD-DNA or WZ-DNA (5 μM) was added per 1 ml of extruded-POPC vesicles (1 $mg \cdot ml^{-1}$).

To ensure efficient incorporation of the DNA in the nanoSLBs and vesicles, the solutions were vortexed and placed at room temperature for ~ 6 hrs, after which they were stored at 4 ℃ until use.

Dynamic light scattering experiments

The size of all investigated samples in solution was examined using dynamic light scattering (DLS) on Zetasizer Nano ZS (Malvern Panalytical, UK). The instrument was equipped with a 50 mW laser with 532 nm wavelength. The intensity correlation function was analyzed using the built-in methods provided by the instrument software, namely distribution fit and cumulant analysis. In the case of monomodal and monodisperse samples, the two methods provide very similar results in terms of average size (called Z_{avg} for the cumulant analysis) and polydispersity index (PDI). In addition, the distribution fit provides an intensity-weighted size distribution histogram as the one shown in Figure S1. It is worth noting that the average size and the PDI reported in the manuscript were obtained by the cumulant analysis, while the size distributions shown in Figure S1 were obtained by the distribution fit. In all cases, the results from the two methods were in qualitative agreement.

QCM-D experiments

The QCM-D experiments were conducted on a QSense E4 system (Biolin, Sweden) on AT-cut 5 MHz quartz sensors, coated with SiO_2. The sensors were cleaned by bath sonication for 15 min in 2%wt. SDS solution, followed by rinsing with Milli-Q water, then drying under a N_2 flow, and finally treating with O_2 plasma for 3 min directly before use. All experiments were conducted at a temperature of 25 ℃. The QCM-D experiments were designed to mimic the flow conditions of the NR experiments, unless otherwise mentioned, solutions with adsorbate molecules were injected on top of the QCM-D crystal using manual syringes or using a peristaltic

pump at 500 μl · min^{-1} after which the flow was stopped and adsorption took place under stagnant flow conditions. QCM-D experiments were conducted to verify the successful formation of the nanoSLBs, optimize the NR experimental conditions, and rule out any non-specific binding of the DNA-decorated nanoSLBs or vesicles.

Neutron reflectometry experiments

Neutron reflectometry (NR) experiments[2] were performed on the INTER reflectometer (ISIS, UK) at the solid-liquid interface,[3] using silicon single crystals as solid substrates (8×5 cm^2 surface, 1.5 cm thick, cut along the 111 plane, polished with a 2 Å RMS nominal roughness). Before the start of each experiment, the silicon crystals were thoroughly cleaned by bath sonication for 15 min in 2%wt. SDS solution, followed by rinsing with Milli-Q water, then drying under a N_2 flow, and finally treating in a UV/Ozone chamber for 45 min directly before use. The crystals were mounted in solid-liquid flow cells directly in liquid to avoid the entrapment of air bubbles at the surface of the crystal. The solid-liquid flow cells were provided by the Institut Laue-Langevin (ILL, Grenoble, France). The polished face of the silicon substrates was kept in contact with a water reservoir throughout sample deposition and measurements by using a PEEK flow module. The module was equipped with inlet and outlet valves for solution injections and exchange. Sample temperature was controlled by means of a circulating water bath, through two aluminum plates put in thermal contact with the silicon block and with the PEEK module; the temperature was maintained at 25.0 ± 0.1 °C for all experiments. Further details about the solid-liquid setup used are given in ref. 4.

NR measurements were performed in time-of-flight mode using wavelengths, λ, from 1.5 to 16 Å, together with two angular configurations, using $\theta = 0.7°$ and $\theta = 2.3°$. In a specular reflectometry experiment, the Q_z vector represents the component of the exchanged wave-vector Q perpendicular to the interface and is expressed as

$$Q_z = \frac{4\pi}{\lambda}\sin(\theta). \tag{S1}$$

With the chosen λ spectrum and θ values, the Q_z-range covered during the measurements was ~ 0.009 Å^{-1} to ~ 0.3 Å^{-1}. Time-of-flight data were converted to reflectivity curves $R(Q_z)$ using MANTID[5] and analyzed using the Aurore software (version 6.0)[6]. In the rest of the manuscript, Q_z is shortly referred to as Q.

Five NR experiments were conducted (Figure S2). Data were collected systematically and fitted after each adsorption step for all experiments. Moreover, the contrast variation method[7] was applied by changing the scattering length density (SLD or ρ) of the aqueous buffer with up to four different D_2O:H_2O mixing ratios after each adsorption step: 100:0 (D_2O; targeted SLD = 6.35×10^{-6} Å^{-2}), 61:39 (SiO_2MW; targeted SLD = 3.47×10^{-6} Å^{-2}), 40:60 (SiMW; targeted SLD = 2.07×10^{-6} Å^{-2}), and 0:100 (H_2O; targeted SLD = -0.56×10^{-6} Å^{-2}). The mixing D_2O ratios for SiO_2MW and SiMW were deliberately chosen slightly higher (2–3%) than the theoretically expected values to compensate for the effect of any H atoms in the D_2O-based buffer, added when preparing the buffer (as HCl was used) and potentially due to exchange with H atoms in the ambient air. The use of the contrast variation method allows not only to increase the structural resolution otherwise limited by the restricted Q-range, but also to reduce the number of possible models able to reproduce a single NR dataset.

Data fitting of the neutron reflectometry data

Collected NR data were sequentially and systematically fitted after each adsorption step to accurately estimate the set of descriptive parameters defining the various layers within a given system. The analysis was performed using modeled NR curves, $R_m(Q)$, which was computed based on the SLD profiles. The latter was calculated by either using a simple slab model or a volume fraction profile (VFP) model, as detailed later. Optimization of the model parameters was achieved by a least-square minimization using the libraries provided within the MINUIT package.[8] The likelihood parameter, χ^2, was used to assess the goodness of fit based on the optimized parameters:

$$\chi^2 = \frac{1}{N_p - N_f}\sum_{j=1}^{N_p}\left[\frac{R(Q_j) - R_m(Q_j)}{\epsilon_j}\right]^2, \tag{S2}$$

where j represents the data points; N_p and N_f are the number of data points and of free parameters, respectively; $R(Q_j)$ and $R_\mathrm{m}(Q_j)$ are the values of experimental and modeled reflectivity at point j; ϵ_j is the absolute experimental error at point j. The minimization routine provided information on the accuracy with which the values of the optimized parameters were determined. Parameters accuracy reported in the manuscript represents 1 standard deviation confidence interval. Parameters kept fixed during the modeling, or computed from quantities known with high accuracy, have null absolute error. During all analyses, the values of the solution SLD were allowed to vary to account for small differences between the nominal solution composition and the real one that originated during the mixing and injection processes.

Bare crystal surfaces and pristine SLBs

NR data for the bare crystal surfaces and POPC SLBs were modeled using the common slab model,[6] assigning one slab to each of the following sample layers: silicon oxide, water gap between the silicon oxide layer and the SLB, SLB inner headgroups, inner tails, outer tails, and outer headgroups. Each slab, i, was characterized by 4 parameters, i.e., thickness, t_i, interfacial roughness, $\sigma_{i,i+1}$, volume fraction occupied by the aqueous solution, $f_{\mathrm{w},i}$, and SLD of the *dry* components, ρ_i. Fitting parameters for the silicon oxide layer were determined for each surface before the SLB deposition, based on NR data acquired in D_2O- and H_2O-based buffers. The obtained parameters were then kept fixed during the modeling of the SLB data. All SLB data were analyzed using the lipid bilayer plug-in available in the Aurore software as described in ref. 6. Briefly, the analysis assumes a symmetric structure of the SLB, i.e., the inner and outer leaflets of the SLB share the same set of structural parameters. Additionally, the model takes into consideration the molecular information about the lipids and applies mathematical constraints in order to reduce the number of free parameters, achieved by establishing a chain of mathematical dependencies between different structural parameters as described in ref. 6. Moreover, within a given SLB, the value of roughness parameters were set to be equal across all the heads and tails layers, and the SLD values of the headgroups and tails of POPC were fixed to $\rho = \frac{b}{V}$, where b and V are the scattering lengths and the molecular volumes of the different portions of the POPC molecule. According to ref. 9: molecular volume V_POPC = 1252 Å^3, PO tail molecular volume V_T = 920.5 Å^3, and PC headgroup molecular volume $V_\mathrm{H} = V_\mathrm{POPC} - V_\mathrm{T}$ = 331.5 Å^3. The coherent scattering lengths of POPC headgroup and tail are b_H = 60.09 fm and b_T = −19.14 fm, respectively, as calculated from the nuclear composition of the two portions of the POPC molecule. Consequently, the SLD of each layer, $\rho_{\mathrm{TOT},i}$, was calculated, taking into account the volume fraction occupied by the solution, according to

$$\rho_{\mathrm{TOT},i} = f_{\mathrm{w},i}\rho_\mathrm{w} + (1 - f_{\mathrm{w},i})\rho_i, \tag{S3}$$

where ρ_w is the SLD of the utilized aqueous solution. Equation S3 allowed to simultaneously model NR data originated from the same *dry* sample exposed to buffer solutions prepared at different H_2O:D_2O concentrations.

SiO_2 NPs and nanoSLBs

In order to properly account for the geometry of the particles, their volume fraction profiles were computed numerically following a similar approach to the one used in ref. 10. VFPs were calculated by assuming that each individual particle was enclosed in a cubic box having sides equal to the particle size (diameter). The bottom face of the box was assumed to be parallel to the solid substrate, and the system was designed to allow for a monolayer of particles, with the bases of all cubic volumes aligned at the same z-value. These two assumptions were necessary to facilitate the volume fraction calculations along the vertical axis perpendicular to the surface, z. For both the SiO_2 NPs and the nanoSLBs, the 1D projection of the volume occupied by particle material was calculated as a function of the position along the z-axis. The free volume in the box was filled with the aqueous solution. For mono-dispersed hard spheres, this situation corresponds to the thinnest regular packing condition, i.e., with centers of spheres arranged on a square lattice,[11] which is characterized by a total particle volume fraction of 0.5236. This type of packing was selected because it allows for easy and fast numerical computations. This packing configuration allows modeling systems with surface coverage up to 78.5%, which is far beyond what is expected in our systems or any known biological system. However, if needed, different packing conditions are also possible. The most relevant is for spheres arranged on a hexagonal lattice, which corresponds to the maximum possible packing of spheres in a two-dimensional plane without overlapping, attaining a surface coverage of 90.7%. This configuration can be modeled by assuming that each individual

NP was enclosed in a hexagonal prism with a side length of $2r_{\mathrm{NP}}/\sqrt{3}$ and a height of $2r_{\mathrm{NP}}$, where r_{NP} is the radius of the NP.

Once obtained, the 1D VFPs were divided into a large number (1000 in the present cases) of thin slabs as an input to calculate the theoretical SLD profiles and reflectivity curves. During the data analysis, the parameters describing the VFPs were modified and optimized to produce reflectivity curves that match as close as possible the collected experimental data.

SiO_2 NPs. SiO_2 NPs were treated as homogeneous solid spheres with a negligible size polydispersity as determined using DLS (Figure S1) and previously determined using atomic force microscopy [12]. Four variables were fitted in order to reproduce the experimentally measured reflectivity, namely: the separation distance between the SLB outer surface and the bottom pole of the NPs, t_{gap}, the NPs radius, r_{NP}, the SLD of the NPs, ρ_{NP}, and the solution volume fraction $f_{\mathrm{w,NP}}$. The separation distance, t_{gap}, was modeled as a single slab with the aqueous solution occupying 100% of its volume and with variable interfacial roughness defined by the neighboring layers. Thus, the lower boundary of the gap layer was defined as the inflection point of VFP of the outer headgroups of the SLB layer, and the upper boundary was defined as the first non-zero value of the VFP of the SiO_2 NP layer. It is worth noting that the inflection point is concurrent with the half maximum of the error function representing the roughness of the headgroup layer.

Numerically, the VFP of the NPs was computed as follows. The sphere axis (along z) with the length of $2r_{\mathrm{NP}}$, was divided into 1000 slabs of equal thickness, namely Δz, which represents the perpendicular distance between two circles produced by cutting a sphere with a pair of parallel planes perpendicular to the z-axis. [13] The radius, r, of each circle is defined according to

$$r(\hat{z}) = \sqrt{2r_{\mathrm{NP}}\hat{z} - \hat{z}^2}, \tag{S4}$$

where $\hat{z}$ is the distance along the z-axis perpendicular to the surface of the silicon block with $\hat{z} = 0$ at the lower pole of the NP, i.e., the pole close to the silicon block. The volume of such spherical slabs can be computed as

$$V_{\mathrm{slab}}(\hat{z}) = \frac{\pi\Delta z}{6}\left(3\left[r(\hat{z}-\Delta z)\right]^2 + 3\left[r(\hat{z})\right]^2 + (\Delta z)^2\right), \tag{S5}$$

where $r(\hat{z}-\Delta z)$ and $r(\hat{z})$ are the radii at $\hat{z}-\Delta z$ and $\hat{z}$, respectively. The 1D volume fraction profile for the case of maximum coverage was then calculated from the ratio between V_{slab} and the volume of the right rectangular prism containing each spherical slab as

$$\upsilon_{\mathrm{NP,max}}(\hat{z}) = \frac{V(\hat{z})}{4r_{\mathrm{NP}}^2\Delta z}. \tag{S6}$$

Figure S3a shows the $\upsilon_{\mathrm{NP,max}}(\hat{z})$, i.e., the case of the maximum packing allowed by the model. If one were to use a model based on spheres enclosed in hexagonal prisms, Equation S6 would have been $\upsilon_{\mathrm{NP,max}}(\hat{z}) = \frac{V(\hat{z})}{2\sqrt{3}r_{\mathrm{NP}}^2\Delta z}$. For reference, the corresponding VFP is shown in Figure S3b.

The conversion from $\upsilon_{\mathrm{NP,max}}(\hat{z})$ to $\rho_{\mathrm{NP}}(\hat{z})$ and the calculation of the theoretical reflectivity curves were performed by first scaling $\upsilon_{\mathrm{NP,max}}(\hat{z})$ to allow more dilute conditions, expressed as

$$\upsilon_{\mathrm{NP}}(\hat{z}) = (1 - f_{\mathrm{w,cube}}) \times \upsilon_{\mathrm{NP,max}}(\hat{z}), \tag{S7}$$

where $f_{\mathrm{w,cube}}$ is the volume fraction of the aqueous solution surrounding the enclosing cubic boxes. The VFP of the total aqueous solution in the NP layer, including the solution within the cubic boxes, is thus equivalent to

$$f_{\mathrm{w,NP}}(\hat{z}) = 1 - \upsilon_{\mathrm{NP}}(\hat{z}). \tag{S8}$$

Finally, the SLD profiles were then calculated according to

$$\rho_{\mathrm{TOT,NP}}(\hat{z}) = f_{\mathrm{w,NP}}(\hat{z}) \times \rho_{\mathrm{w}} + \left[1 - f_{\mathrm{w,NP}}(\hat{z})\right] \times \rho_{\mathrm{NP}}. \tag{S9}$$

During multiple contrasts analysis, multiple SLD profiles and NR curves were generated by the same VFP by using the appropriate solution SLD values ρ_{w} in Equation S9.

nanoSLBs. The gap layer for the nanoSLBs was modeled in a similar fashion to that of the SiO_2 NPs, with one minor difference. Unlike the SiO_2 NPs, in which the gap layer is completely filled with solution, the gap layer for the nanoSLBs contains the linking DNA molecules. To account for this difference, the gap layer was fitted for both t_{gap} and $f_{\text{w,gap}}$, while fixing the SLD to $\rho_{\text{gap}} = 2\times10^{-6}\,\text{Å}^{-2}$, the expected SLD value for DNA. For both nanoSLB samples, $f_{\text{w,gap}}$ was larger than 0.99, indicating that the gap layer is mostly filled by solution. Additionally, while the SiO_2 NPs are single-component particles, nanoSLBs are composed of a SiO_2 core and a SLB shell, i.e., components with different SLD values. For sufficiently accurate calculations of reflectivity, the VFPs from both the SiO_2 core and the SLB shell have to be calculated separately in the model. Thus, the parameters describing a nanoSLB layer are the separation distance, t_{gap}, the core radius, r_{core}, the core SLD, ρ_{core}, the shell thickness, t_{shell}, the shell SLD, ρ_{shell}, and the solution volume fraction, $f_{\text{w,nanoSLB}}$. The SiO_2 core VFP at maximum coverage ($v_{\text{core,max}}$) was calculated using similar procedure to the one described for the SiO_2 NPs (Equations S4–S6), but with three differences: (i) for consistency, r_{NP} was replaced with r_{core}; (ii) the enclosing cubic box had a side = $2(r_{\text{core}} + t_{\text{shell}})$; (iii) $\hat{z} = 0$ was at lower pole of the nanoSLBs, thus $\hat{z}$ in Equations S4 and S5 were replaced with $\hat{z} - t_{\text{shell}}$. The SLB shell VFP at maximum coverage ($v_{\text{shell,max}}$) was calculated as the difference between the VFP of a homogeneous sphere of radius $r_{\text{core}} + t_{\text{shell}}$, using Equations S4–S6, and the VFP of the core sphere. It is worth noting that, contrary to the case of planar SLBs, the bilayer internal structure is not visible because of the orientational averaging performed in calculating the 1D VFP. The total maximum VFP of the nanoSLB layer was obtained as $v_{\text{nanoSLB,max}} = v_{\text{core,max}} + v_{\text{shell,max}}$ (Figure S4). Based on which, under diluted conditions, the VFPs were calculated according to

$$\begin{aligned} v_{\text{core}}(\hat{z}) &= (1 - f_{\text{w,cube}}) \times v_{\text{core,max}}(\hat{z}) \\ v_{\text{shell}}(\hat{z}) &= (1 - f_{\text{w,cube}}) \times v_{\text{shell,max}}(\hat{z}) \\ v_{\text{nanoSLB}}(\hat{z}) &= v_{\text{core}}(\hat{z}) + v_{\text{shell}}(\hat{z}), \end{aligned} \tag{S10}$$

where $f_{\text{w,cube}}$ is the volume fraction of the aqueous solution surrounding the enclosing cubic boxes. The VFP of the total aqueous solution in the nanoSLBs layer, including the contribution within the cubic boxes, is thus equivalent to

$$f_{\text{w,nanoSLB}}(\hat{z}) = 1 - v_{\text{nanoSLB}}(\hat{z}). \tag{S11}$$

Finally, the SLD profiles can be calculated according to

$$\rho_{\text{TOT,nanoSLB}}(\hat{z}) = f_{\text{w,nanoSLB}}(\hat{z}) \times \rho_{\text{w}} + v_{\text{core}}(\hat{z}) \times \rho_{\text{core}} + v_{\text{shell}}(\hat{z}) \times \rho_{\text{shell}}. \tag{S12}$$

Vesicles. The vesicles layer was modeled using a single slab featuring large asymmetric roughness: $\sigma_{\text{gap,vesicle}}$ at the interface between the separation gap and the bottom pole of the vesicles layer, and $\sigma_{\text{vesicle,bulk}}$ between the vesicles and the bulk solution. The other slab parameters were its thickness (t_{vesicle}), SLD (ρ_{vesicle}), and solution volume fraction ($f_{\text{w,vesicle}}$). This model was used because accounting for the polydispersity of vesicles in the VFP model came at a high computational cost. The VFP profile obtained by this model was then used as a reference to fit a theoretical VFP computed, assuming that all vesicles remained spherical after attachment (an approximating assumption sustained by the relatively small size of the vesicles) and that their size distribution followed a Schulz distribution (Equation S13) [14]

$$f(r) = (w+1)^{(w+1)} \left(\frac{r}{r_{\text{avg}}}\right)^{w} \frac{e^{-(w+1)\frac{r}{r_{\text{avg}}}}}{r_{\text{avg}}\Gamma(w+1)}, \tag{S13}$$

where r is the core radius of the vesicles, r_{avg} is the average core radius, w reflects the width of the distribution, and Γ is the gamma function. The Schulz distribution was selected as it is non-null only for positive values of r with $f(0) = 0$. In turn, the average vesicles size was determined as $\tilde{\phi} = 2(r_{\text{avg}} + t_{\text{shell}})$; t_{shell} was assumed to be 4.1 nm, which is equivalent to the fitted thickness of the shells of the ABCD-nanoSLBs (also in agreement with refs. 9,15,16). The theoretical VFP was thus calculated as

$$v_{\text{vesicle}} = s \times \sum_{r} f(r) v_{\text{shell}}(r), \tag{S14}$$

where $v_{\text{shell}}(r)$ represents the VFP of a vesicle with a core radius of r in a cubic box with side $2(r_{\text{avg}} + t_{\text{shell}})$ and s is a scaling factor equal to the maximum value of the VFP extracted from the SLD profile resulting from the slab model. Equation S14 was used to reproduce the shape of the VFP determined from the slab model analysis of NR data by calculating v_{vesicle} for r = 0–100 nm. During this procedure, the fitting parameters were r_{avg} and w. A typical result of this refinement procedure is shown in Figure S5.

The use of a slab model to describe the vesicles layer has an indirect effect on the thickness evaluation of the underlying gap layer, which is equal to the distance between the inflection points of the error functions used to model the interfacial roughness of the outer headgroups of the planar SLB and the interfacial roughness of the vesicles layer at it lower pole, i.e., the one closer to the Si block. However, the slab model used to describe the vesicles layers treats the non-constant volume fraction observed at the lower pole of the vesicles with a height of t_{shell} (Figure S5a) as roughness at the interface, thus resulting in an overestimation of the gap thickness by a value of ~ $t_{\text{shell}}/2$. On the contrary, for the SiO_2 NPs and the nanoSLBs, the VFP-model takes into account the non-constant volume fraction of the particles through the layer, precisely defining the gap/particle interface at the lower pole of the particles. To overcome this difference, we define t_{gap} for the vesicle layers as:

$$t_{\text{gap}} = t_{\text{gap,slab}} - t_{\text{shell}}/2, \tag{S15}$$

where $t_{\text{gap,slab}}$ is the fitted thickness of the gap layer and t_{gap} is the actually gap distance between the planar SLB and the lower pole of the vesicles.

Surface coverage calculations

Surface coverage for the SiO_2 NPs and nanoSLBs were taken to be equal to the maximum value of their VFPs, i.e., the volume fraction at $\hat{z} = r_{\text{NP}}$ and $\hat{z} = r_{\text{core}} + t_{\text{shell}}$ for SiO_2 NPs and nanoSLBs, respectively. This was possible due to their narrow size distribution. i.e., the maximum cross-sectional area of all the particles in each system occurs at the same perpendicular distance from the surface. In the case of polydispersed vesicles, one could use the average size of the vesicles to estimate the surface coverage; however, this would result in an inaccurate estimation. We have therefore calculated the surface coverage taking into account the size distribution as follows. First, an equivalent cross-sectional area was calculated according to

$$A_{\text{vesicle}} = \sum_r f(r) \times \pi(r + t_{\text{shell}})^2, \tag{S16}$$

where $f(r)$ is defined according to Equation S13. The surface coverage would then be equal to $n \times A_{\text{vesicle}}$, where n is the number of vesicles per unit area. We thus used the maximum value of the experimentally determined volume fraction of the vesicle to determine n. To this end, and in a similar manner to the cross-sectional area, we calculate the equivalent shell volume as

$$V_{\text{shell}} = \sum_r f(r) \times g(r), \tag{S17}$$

where $g(r)$ is a function that calculates the shell volume of a 1 Å thick slab; it is worth noting that the volume and thus the volume fraction of all slabs with a fixed thickness between $\hat{z} = t_{\text{shell}}$ and $\hat{z} = t_{\text{shell}} + 2r_{\text{core}}$ are constant (Figure S4). Consequently, n was calculated by dividing the maximum value of the VFP by V_{shell}, and the surface coverage was calculated as $n \times A_{\text{vesicle}}$. The approach is graphically illustrated in Figure S6.

Table S1: Values of the parameters describing the silicon oxide and SLB layers obtained from fitting the NR data. One standard-deviation errors were calculated by the MINUIT algorithm from the second derivative of the χ^2 function as described in Ref. 6.

**Parameter**	**Exp. #1**		**Exp. #2**		**Exp. #3**		**Exp. #4**		**Exp. #5**		
	Value	Error	Value	Error	Value	Error	Value	Error	Value	Error	Units
σ_{Si}	2.0	fixed	2.0	fixed	2.0	fixed	2.0	fixed	2.0	fixed	Å
$t_{\mathrm{SiO_x}}$	12.6	0.5	8.8	0.6	10	1	7.7	0.9	11.3	0.7	Å
$\rho_{\mathrm{SiO_x}}$	3.41	fixed	3.41	fixed	3.41	fixed	3.41	fixed	3.41	fixed	$\times 10^{-6}$ Å^{-2}
$f_{\mathrm{w,SiO_x}}$	0.0	fixed	0.0	fixed	0.0	fixed	0.0	fixed	0.0	fixed	–
$\sigma_{\mathrm{SiO_x}}$	5.5	0.5	3.5	0.7	4.4	0.9	3.4	0.4	4.9	0.6	Å
$t_{\mathrm{gap,1}}$	4.9	0.2	4.9	0.2	5.0	0.2	5.3	0.5	5.8	0.2	Å
t_{H}	6.8	0.1	8.1	0.5	7.0	0.7	6.9	0.6	6.5	0.3	Å
ρ_{H}	1.81	fixed	1.81	fixed	1.81	fixed	1.81	fixed	1.81	fixed	$\times 10^{-6}$ Å^{-2}
$f_{\mathrm{w,H}}$	0.2	fixed	0.3	0.1	0.2	0.1	0.2	0.1	0.17	0.05	–
t_{T}	15.0	0.1	15.2	0.1	15.1	0.1	15.6	0.1	15.0	0.4	Å
ρ_{T}	-0.21	fixed	-0.21	fixed	-0.21	fixed	-0.21	fixed	-0.21	fixed	$\times 10^{-6}$ Å^{-2}
$f_{\mathrm{w,T}}$	0.00	0.01	0.01	0.01	0.00	0.01	0.04	0.05	0.00	0.01	–
t_{SLB}	43.6	0.4	47	1	44	2	45	1	43	1	Å
σ_{SLB}	5.2	0.3	3.5	0.4	5.1	0.4	1.5	0.5	4.8	0.5	Å

Table S2: Values of the parameters describing the SiO_2 NP layer obtained from fitting the NR data. Parameters describing the silicon oxide and SLB layers were kept fixed to the values reported in Table S1, Exp. #1. One standard-deviation errors were calculated by the MINUIT algorithm from the second derivative of the χ^2 function as described in Ref. 6.

Parameter	Value	Error	Units
t_{gap}	10	10	Å
r_{NP}	710	10	Å
ρ_{NP}	3.6	0.2	$\times 10^{-6}$ Å^{-2}
$f_{\mathrm{w,NP}}$	0.63	0.05	–

Table S3: Values of the parameters describing the ABCD-attached nanoSLB layer obtained from fitting the NR data. Parameters describing the silicon oxide and SLB layers were kept fixed to the values reported in Table S1, Exp. #2. One standard-deviation errors were calculated by the MINUIT algorithm from the second derivative of the χ^2 function as described in Ref. 6.

Parameter	Value	Error	Units
t_{gap}	70	10	Å
r_{core}	700	20	Å
ρ_{core}	3.3	0.2	$\times 10^{-6}$ Å^{-2}
t_{shell}	41	5	Å
ρ_{shell}	0.33	0.05	$\times 10^{-6}$ Å^{-2}
$f_{\mathrm{w,nanoSLB}}$	0.47	0.05	–

Table S4: Values of the parameters describing the XYWZ-attached nanoSLB layer obtained from fitting the NR data. Parameters describing the silicon oxide and SLB layers were kept fixed to the values reported in Table S1, Exp. #3. One standard-deviation errors were calculated by the MINUIT algorithm from the second derivative of the χ^2 function as described in Ref. 6.

Parameter	Value	Error	Units
t_{gap}	35	5	Å
r_{core}	730	20	Å
ρ_{core}	3.4	0.2	$\times 10^{-6}$ Å^{-2}
t_{shell}	41	fixed	Å
ρ_{shell}	0.33	0.05	$\times 10^{-6}$ Å^{-2}
$f_{\text{w,nanoSLB}}$	0.79	0.05	0:1

Table S5: Values of the parameters describing the ABCD-attached vesicle layer obtained from fitting the NR data. Parameters describing the silicon oxide and SLB layers were kept fixed to the values reported in Table S1, Exp. #4. One standard-deviation errors were calculated by the MINUIT algorithm from the second derivative of the χ^2 function as described in Ref. 6.

Parameter	Value	Error	Units
$t_{\text{gap,slab}}$	85	5	Å
t_{gap}	65	5	Å
t_{vesicles}	360	10	Å
ρ_{shell}	0.3	0.1	$\times 10^{-6}$ Å^{-2}
$f_{\text{w,vesicles}}$	0.77	0.05	–
$\sigma_{\text{gap,vesicles}}$	30	2	Å
$\sigma_{\text{vesicles,bulk}}$	118	5	Å
t_{shell}	41	fixed	Å
r_{avg}	178	5	Å
w	7.4	0.3	–

Table S6: Values of the parameters describing the XYWZ-attached vesicle layer obtained from fitting the NR data. Parameters describing the silicon oxide and SLB layers were kept fixed to the values reported in Table S1, Exp. #5. One standard-deviation errors were calculated by the MINUIT algorithm from the second derivative of the χ^2 function as described in Ref. 6.

Parameter	Value	Error	Units
$t_{\text{gap,slab}}$	44	9	Å
t_{gap}	23	9	Å
t_{vesicles}	460	20	Å
ρ_{shell}	0.3	0.1	$\times 10^{-6}$ Å^{-2}
$f_{\text{w,vesicles}}$	0.9	0.1	–
$\sigma_{\text{gap,vesicles}}$	27	2	Å
$\sigma_{\text{vesicles,bulk}}$	150	30	Å
t_{shell}	41	fixed	Å
r_{avg}	236	5	Å
w	7.5	0.2	–

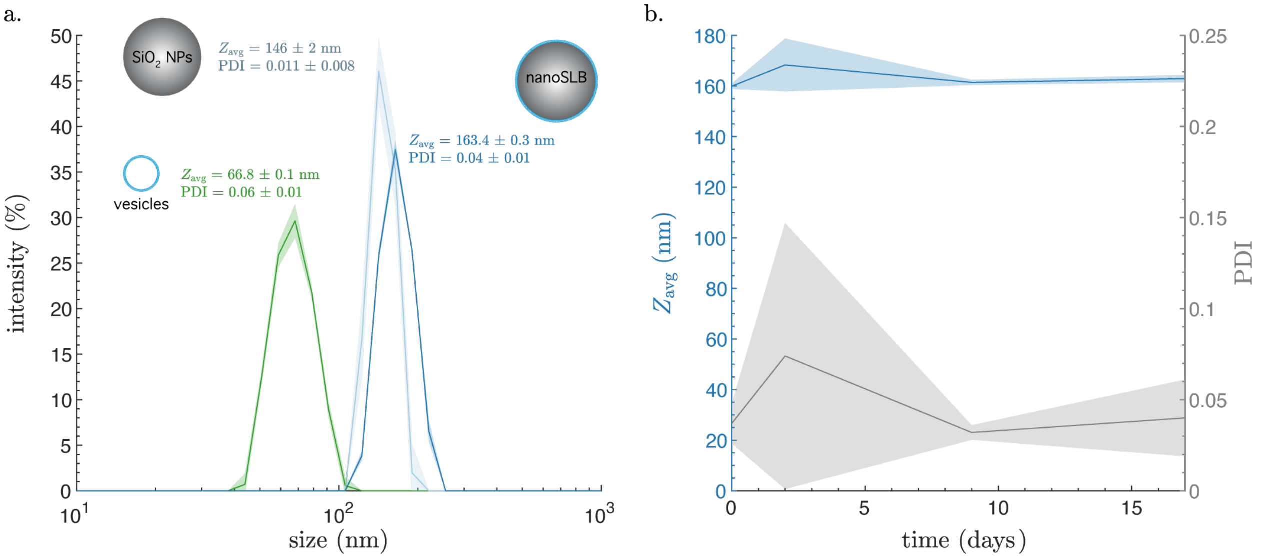

Figure S1: **Size distribution of extruded-POPC vesicles, SiO_2 NPs, and nanoSLBs determined using DLS.** (a) Scattering intensity versus size of extruded-POPC vesicles, SiO_2 NPs, and nanoSLBs as determined using DLS. The Z_{avg} of the nanoSLBs were ~ 17 nm larger than that of the SiO_2 NPs, while retaining a very low polydispersity index (PDI) of 0.04 ± 0.02, only slightly higher than the PDI of the SiO_2 NPs of 0.011 ± 0.008. These results suggest the successful formation of the lipid bilayer coating of the nanoSLBs and also the successful purification of the sample form any residual vesicles; any aggregation of the nanoSLBs or presence of substantial amounts of bound or free unraptured vesicles would have resulted in a higher PDI. The Z_{avg} and PDI of the extruded-POPC vesicles were 66.8 ± 0.1 nm and 0.06 ± 0.01, respectively, suggesting a relatively tight size distribution; in comparison the Z_{avg} and PDI of sonicated-POPC vesicles (data not shown) were 59.1 ± 0.3 nm and 0.177 ± 0.004, respectively. The sonicated-POPC vesicles had smaller but broader size distribution than the extruded-POPC vesicles. It is worth noting that the Z_{avg} values were calculated based on the cumulant analysis and therefore have slightly different values than the distribution fits shown in the panel. (b) The change in Z_{avg} and PDI overtime for the nanoSLBs suggests that they were stable without any substantial aggregation over a period of more than two weeks. All NR experiments were conducted within one week of the preparation of the nanaoSLBs.

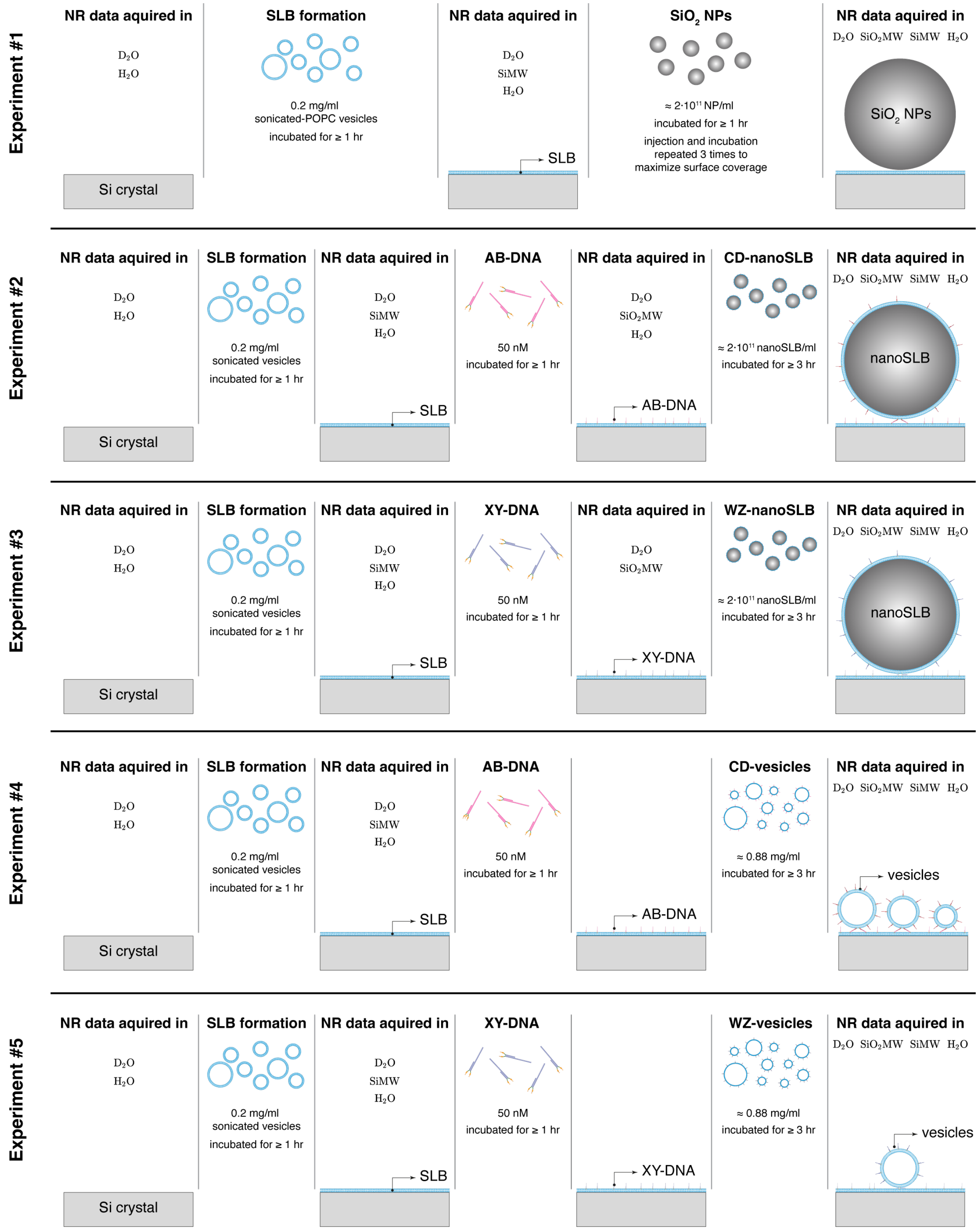

Figure S2: Schematics of the five conducted NR experiments, showing the several steps of each experiment and D_2O:H_2O mixtures in which the NR data were collected. For experiments 4 and 5 no NR data were collected after the AB-DNA and XY-DNA steps, respectively, because results from experiments 2 and 3 showed that the contrast of the added DNA molecules was too small to be detected.

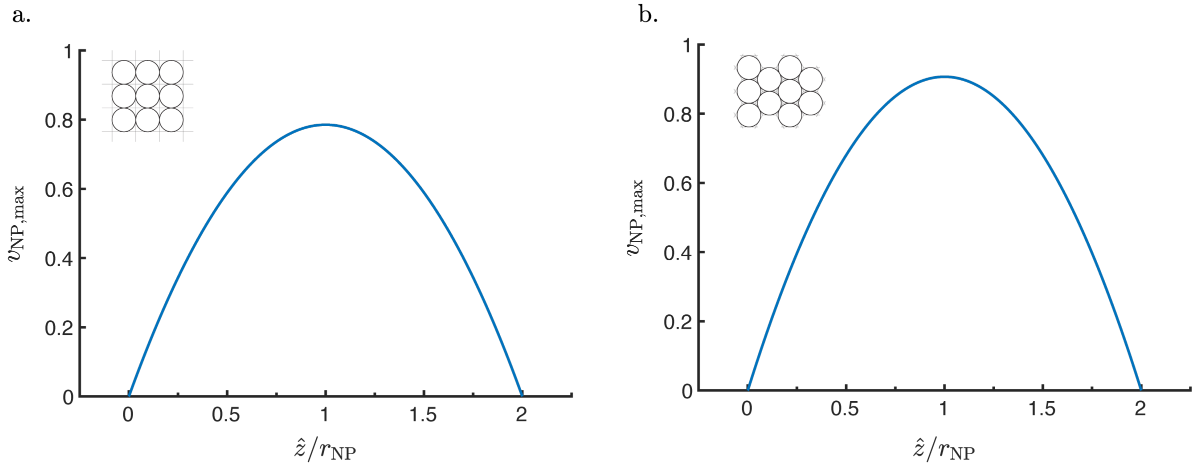

Figure S3: (a) Volume fraction profile ($v_{\mathrm{NP,max}}$) for a layer composed of spheres of radius r_{NP} in the thinnest regular packing condition, i.e., for spheres arranged on a square lattice. The VFP was calculated assuming a sphere enclosed in a cubic box with a side length of $2r_{\mathrm{NP}}$. Using this configuration for fitting the NR data allows for a maximum surface coverage of 78.5%, equivalent to the maximum value of VFP at the equatorial position. (b) Volume fraction profile ($v_{\mathrm{NP,max}}$) for a layer composed of spheres of radius r_{NP} arranged on a hexagonal lattice, i.e., the highest packing density possible for spheres arranged in a 2-dimensional plane without overlapping. The VFP was calculated assuming a sphere enclosed in a hexagonal prism with a side length of $2r_{\mathrm{NP}}/\sqrt{3}$ and a height of $2r_{\mathrm{NP}}$. Using this configuration for fitting the NR data allows for a maximum surface coverage of 90.7%, equivalent to the maximum value of VFP at the equatorial position.

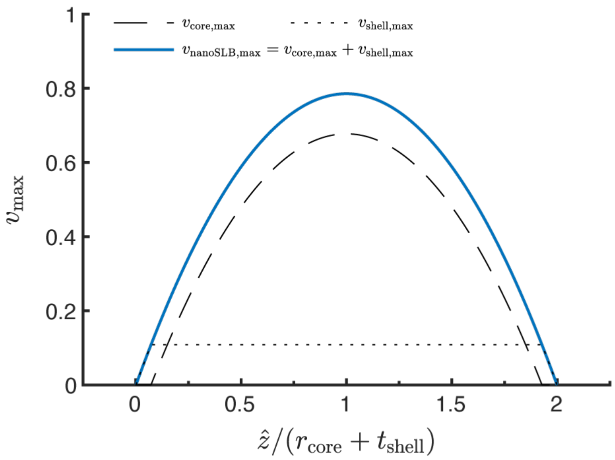

Figure S4: Volume fraction profile ($v_{\mathrm{nanoSLB,max}}$) for a nanoSLB layer, with core radius r_{core} and shell thickness t_{shell} in thinnest regular packing condition, i.e., for spheres arranged on a square lattice.

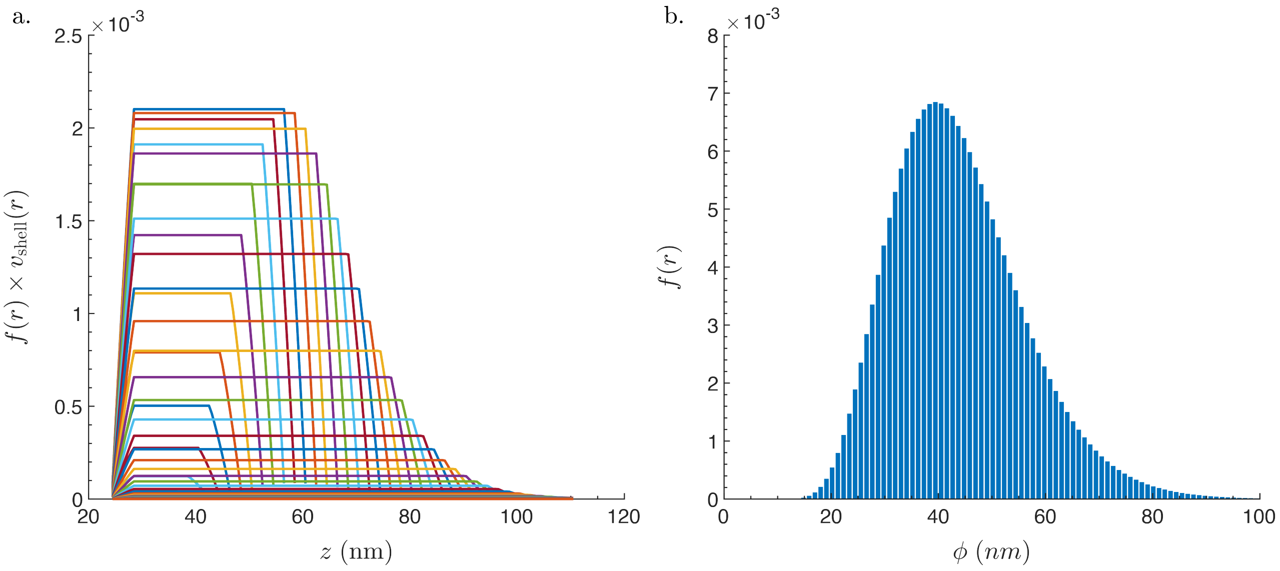

Figure S5: **Volume fraction profiles and size distribution of ABCD-attached vesicles.** (a) Individual $v_{\mathrm{shell}}(r)$ contributions weighted by their frequency of occurrence given by $f(r)$ for the ABCD-attached vesicles (Equation S14). Their sum results in the VFP shown in the inset of Figure 4c. (b) Size distribution obtained from the analysis of the VFP for the ABCD-attached vesicles using Equations S13 and S14.

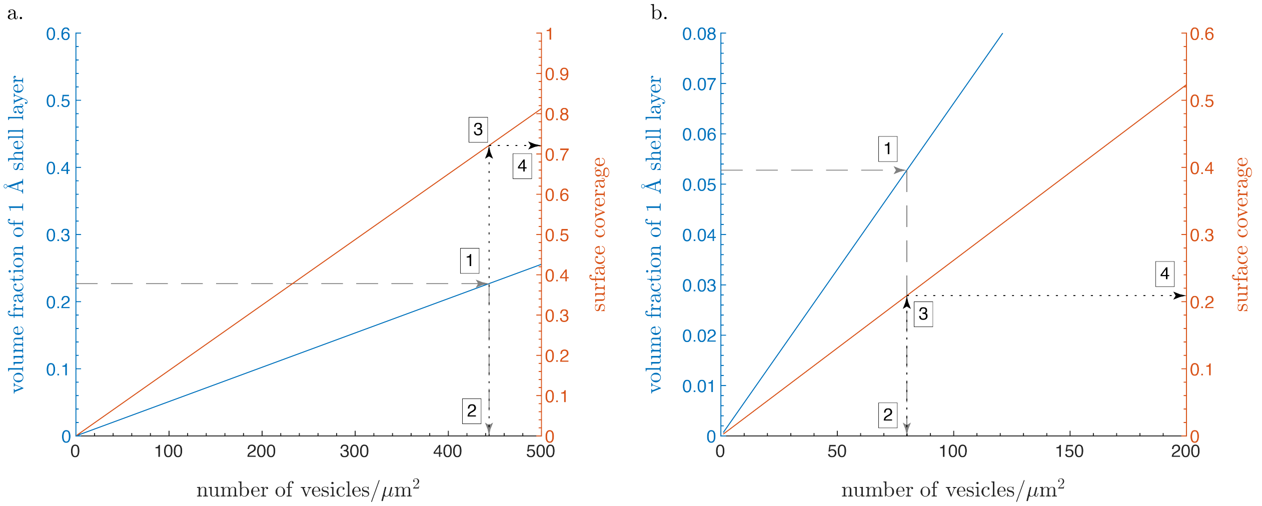

Figure S6: **Surface coverage calculations for the ABCD- and XYWZ-attached vesicles.** Surface coverage calculations for the (a) ABCD- and the (b) XYWZ-attached vesicles were conducted based on the maximum volume fraction value obtained from the fitting of the experimental data and by taking the size distribution of vesicles into consideration as detailed in the Materials and Methods section. The figures show a graphical representation of the calculations: the maximum value of the experimentally fitted VFP was determined, here denoted with the dashed horizontal line ending with the label (1); based on which the corresponding number of vesicles were calculated based on the theoretical relation between the number of vesicles and the volume fraction of the slab ($\equiv n \times V_{\text{shell}}$, where n is number of vesicles and V_{shell} is defined according to Equation S17), here denoted with the dashed vertical line ending with the label (2); the number of vesicles was then used to determine the surface coverage based on the theoretical relation between the number of vesicles and surface coverage ($\equiv n \times A_{\text{vesicle}}$, where A_{vesicle} is defined according to Equation S16), here denoted with the two dotted lines ending with labels (3) and (4)

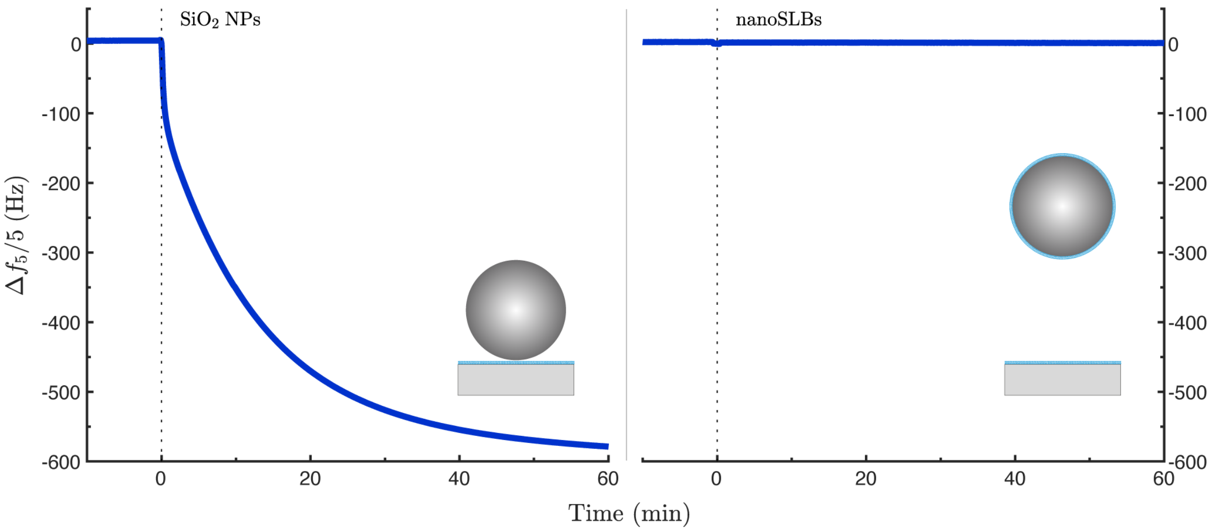

Figure S7: **Adsorption of SiO_2 NPs and nanoSLBs to POPC SLB.** QCM-D frequency response due to the adsorption of SiO_2 NPs and nanoSLBs to planar POPC SLBs. While SiO_2 NPs adsorbed extensively, no detectable adsorption was observed for the nanoSLBs. The results further confirm the successful formation of the lipid bilayer coating on the nanoSLBs. Incomplete or patchy coating of the nanoSLBs would have likely resulted in measurable adsorption of the nanoSLBs to the SLB. Both experiments were conducted at particles concentration of ~ 2×10^{11} particle $\cdot$ ml^{-1} and under stagnant flow conditions.

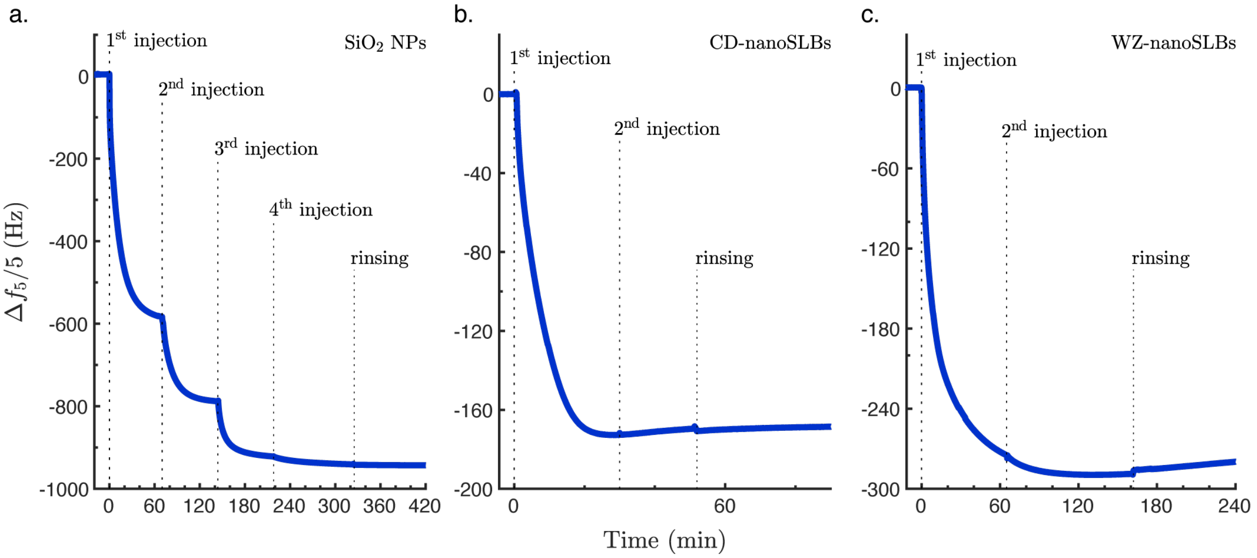

Figure S8: **Optimization of the NR experimental procedure using QCM-D** The frequency response due to several injections of (a) SiO_2 NPs on SLB, (b) CD-nanoSLBs on AB-DNA decorated SLB, (c) WZ-nanoSLBs on XY-DNA decorated SLB. The solutions were injected by hand using a syringe and left under no-flow conditions to mimic the NR experimental conditions. While SiO_2 NPs needed three injections with incubation times of ~ 60 min for the adsorption to plateau, CD-nanoSLBs and WZ-nanoSLBs needed only one injection. These experiments were conducted with the same patch of SiO_2 NPs and nanoSLBs used for the NR experiments.

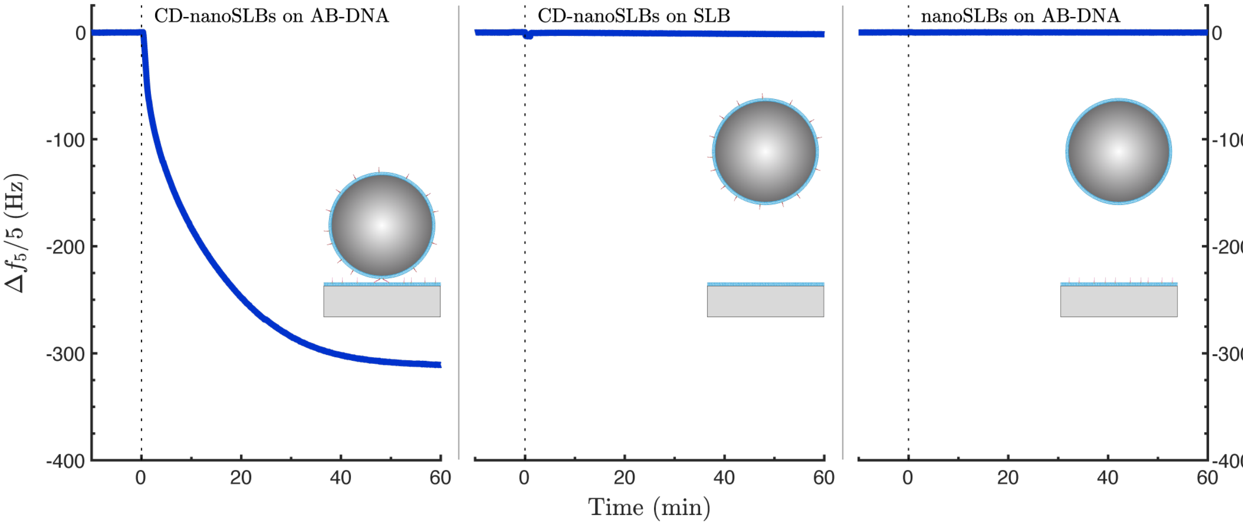

Figure S9: **Specificity of the hybridization between the AB-DNA and the CD-nanoSLBs.** QCM-D frequency response due to the attachment of CD-nanoSLBs to AB-DNA decorated SLB, CD-nanoSLBs to bare SLB, and bare nanoSLBs to AB-DNA decorated SLB. While the CD-nanoSLBs adsorbed extensively to the AD-DNA decorated SLB, no detectable adsorption was observed in the other two cases. The results verify the specificity of the attachment between the CD-nanoSLBs to AB-DNA decorated SLB, and rule out any measurable contribution from non-specific attachment of the nanoSLBs to the SLB. All experiments were conducted at particles concentration of $\sim 2 \times 10^{11}$ particle $\cdot$ ml^{-1} and under stagnant flow conditions.

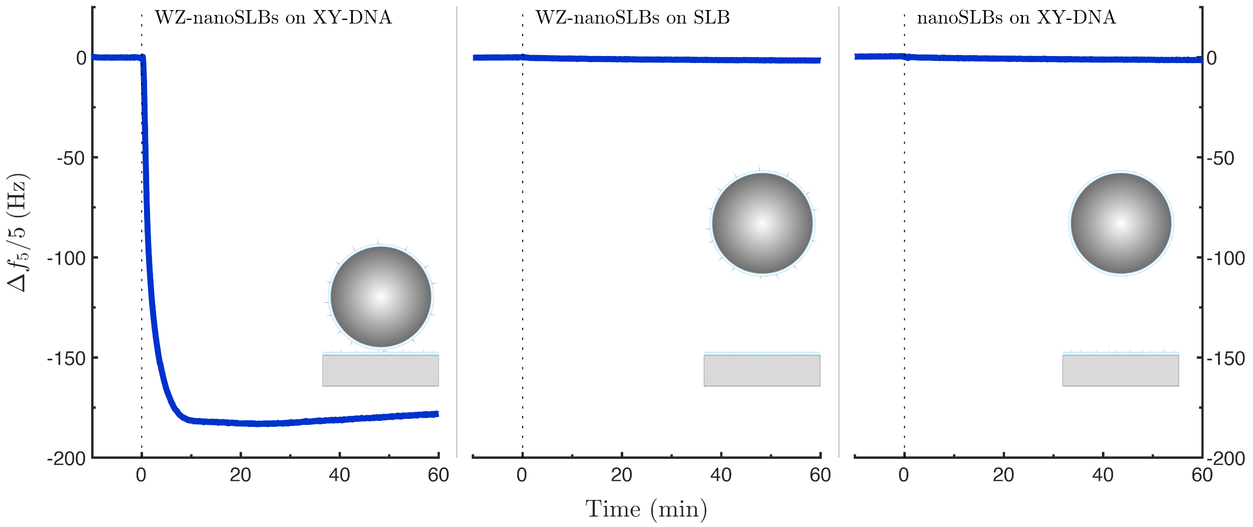

Figure S10: **Specificity of the hybridization between the XY-DNA and the WZ-nanoSLBs.** QCM-D frequency response due to the attachment of WZ-nanoSLBs to XY-DNA decorated SLB, WZ-nanoSLBs to bare SLB, and bare nanoSLBs to XY-DNA decorated SLB. While the WZ-nanoSLBs adsorbed extensively to the XY-DNA decorated SLB, no detectable adsorption was observed in the other two cases. The results verify the specificity of the attachment between the WZ-nanoSLBs to XY-DNA decorated SLB, and rule out any measurable contribution from non-specific attachment of the nanoSLBs to the SLB. All experiments were conducted at particles concentration of ~ 2×10^{11} particle $\cdot$ ml^{-1} and under stagnant flow conditions.

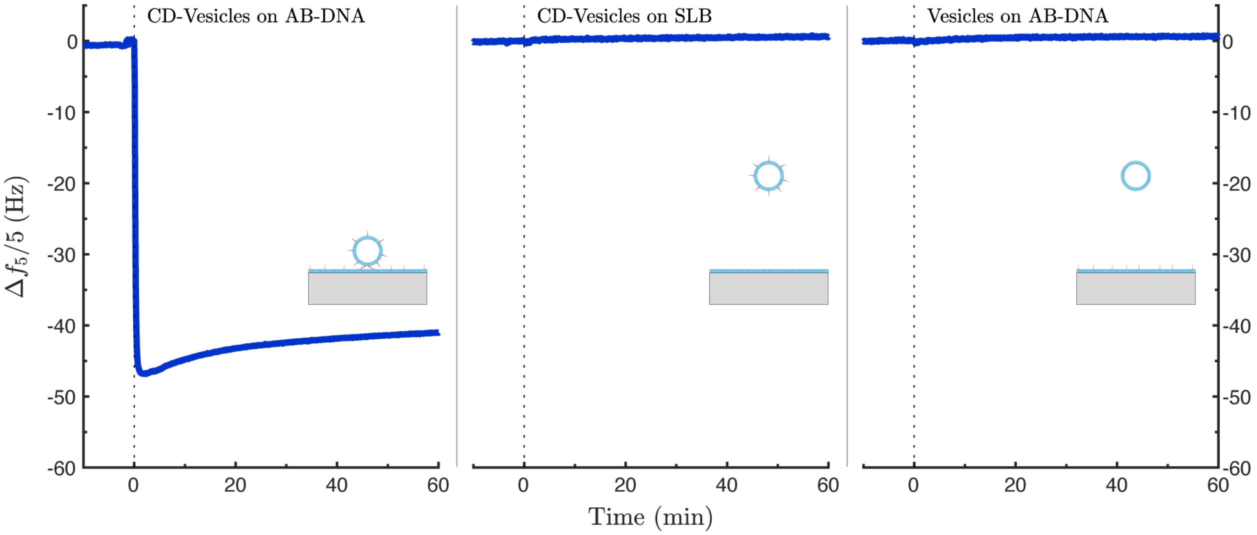

Figure S11: **Specificity of the hybridization between the AB-DNA and the CD-vesicles.** QCM-D frequency response due to the attachment of CD-vesicles to AB-DNA decorated SLB, CD-vesicles to bare SLB, and bare vesicles to AB-DNA decorated SLB. While the CD-vesicles adsorbed extensively to the AB-DNA decorated SLB, no detectable adsorption was observed in the other two cases. The results verify the specificity of the attachment between the CD-vesicles to AB-DNA decorated SLB, and rule out any measurable contribution from non-specific attachment of the vesicles to the SLB. Experiments with CD-vesicles were conducted at a concentration of 0.88 $\mathrm{mg \cdot ml^{-1}}$; the experiment with bare vesicles was conducted at a concentration of 1.0 $\mathrm{mg \cdot ml^{-1}}$. All experiments were conducted under stagnant flow conditions.

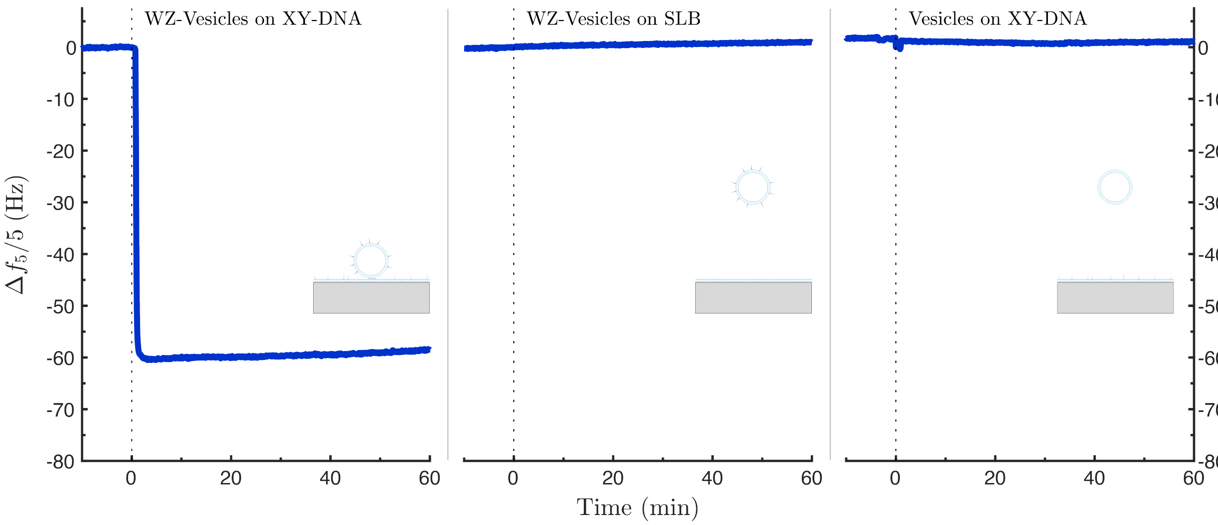

Figure S12: **Specificity of the hybridization between the XY-DNA and the WZ-vesicles.** QCM-D frequency response due to the attachment of WZ-vesicles to XY-DNA decorated SLB, WZ-vesicles to bare SLB, and bare vesicles to XY-DNA decorated SLB. While the WZ-vesicles adsorbed extensively to the XY-DNA decorated SLB, no detectable adsorption was observed in the other two cases. The results verify the specificity of the attachment between the WZ-vesicles to XY-DNA decorated SLB, and rule out any measurable contribution from non-specific attachment of the vesicles to the SLB. Experiments with WZ-vesicles were conducted at a concentration of 0.88 $mg \cdot ml^{-1}$; the experiment with bare vesicles was conducted at a concentration of 1.0 $mg \cdot ml^{-1}$. All experiments were conducted under stagnant flow conditions.

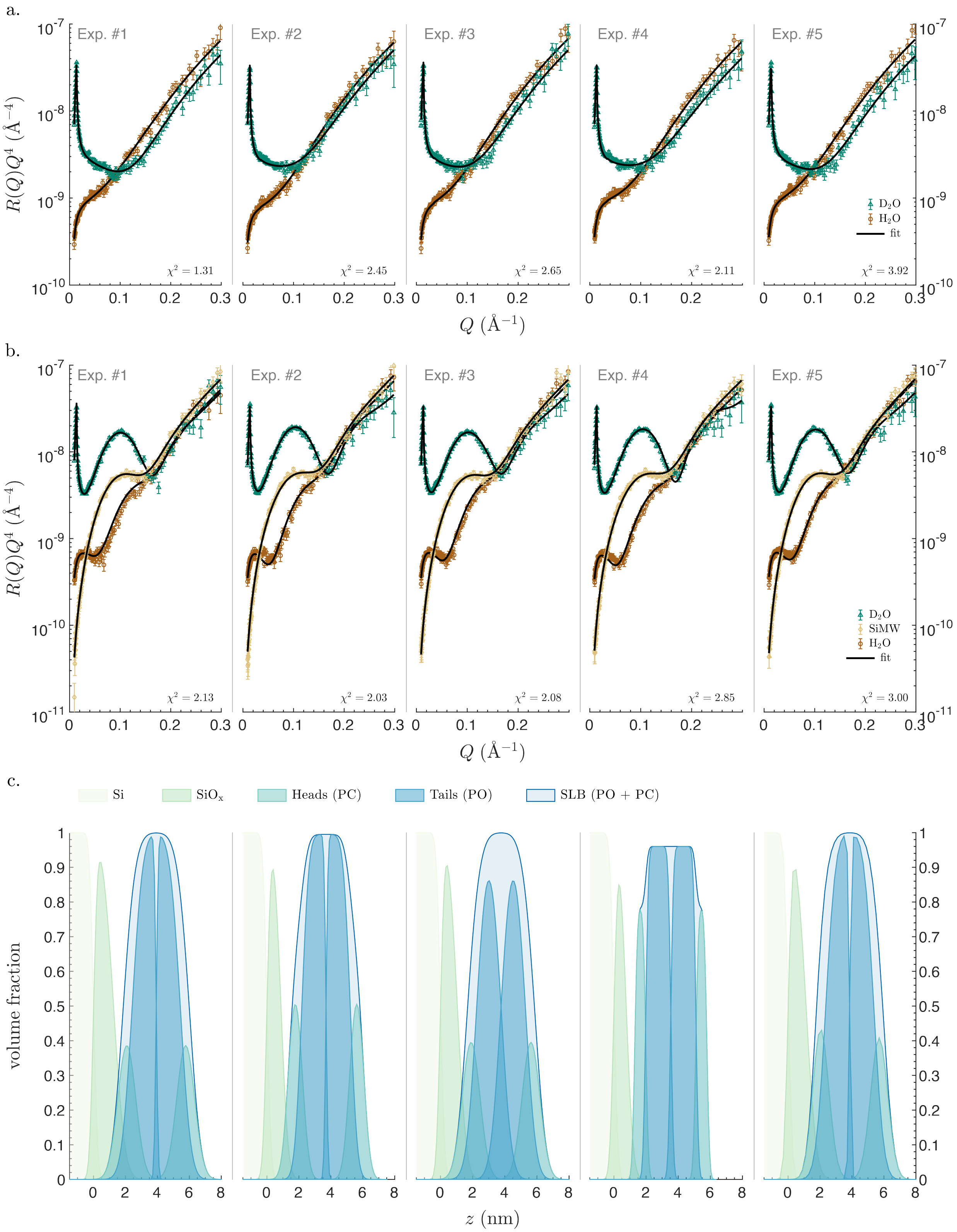

Figure S13: **NR data, fits, and volume fraction profiles for the SiOx and SLB layers for all five experiments.** NR data and fits for (a) the SiOx layers and (b) the SLB layers from all five experiments with the corresponding volume fraction profiles shown in (c). NR data for the SiOx layer were collected in D_2O and H_2O and for the SLB layers in D_2O, SiMW, and H_2O. Differences among the datasets are mostly induced by the variability of the roughness of the solid substrates used.

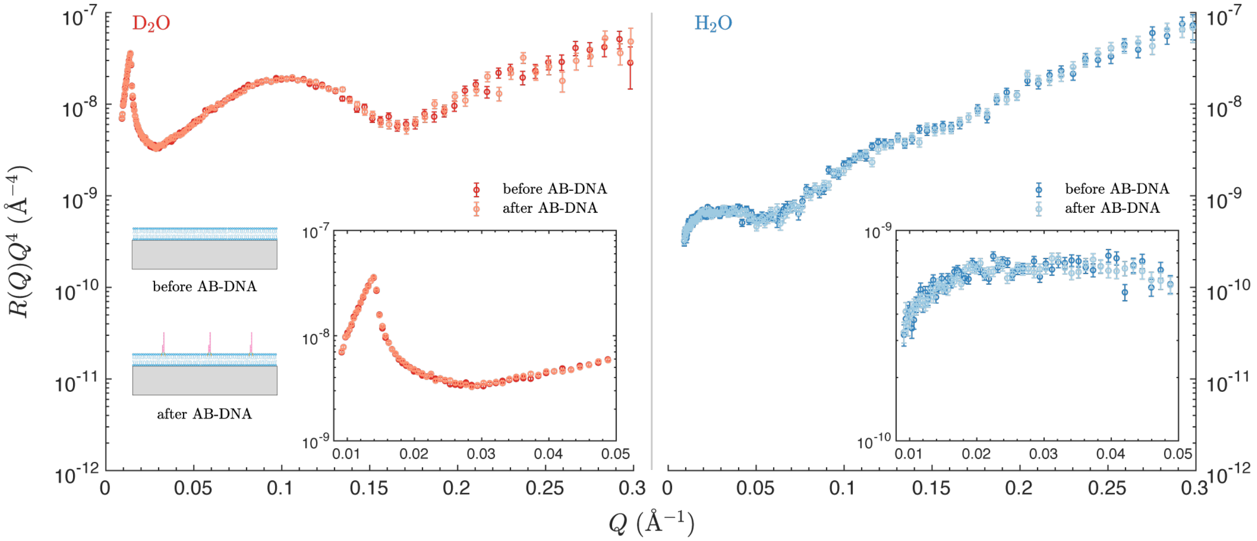

Figure S14: **NR data before and after the AB-DNA adsorption step.** No detectable difference in the NR data was observed before and after the AB-DNA adsorption step; these results indicate that the presence of the AB-DNA did not perturb the SLB structure and that they were diluted, having a low volume fraction below the detection limit. These results are consistent with the QCM-D results, which showed ≤ 2 Hz response due to the adsorption of the AB-DNA molecules. Similar results were obtained for the XY-DNA in D_2O (data not shown).

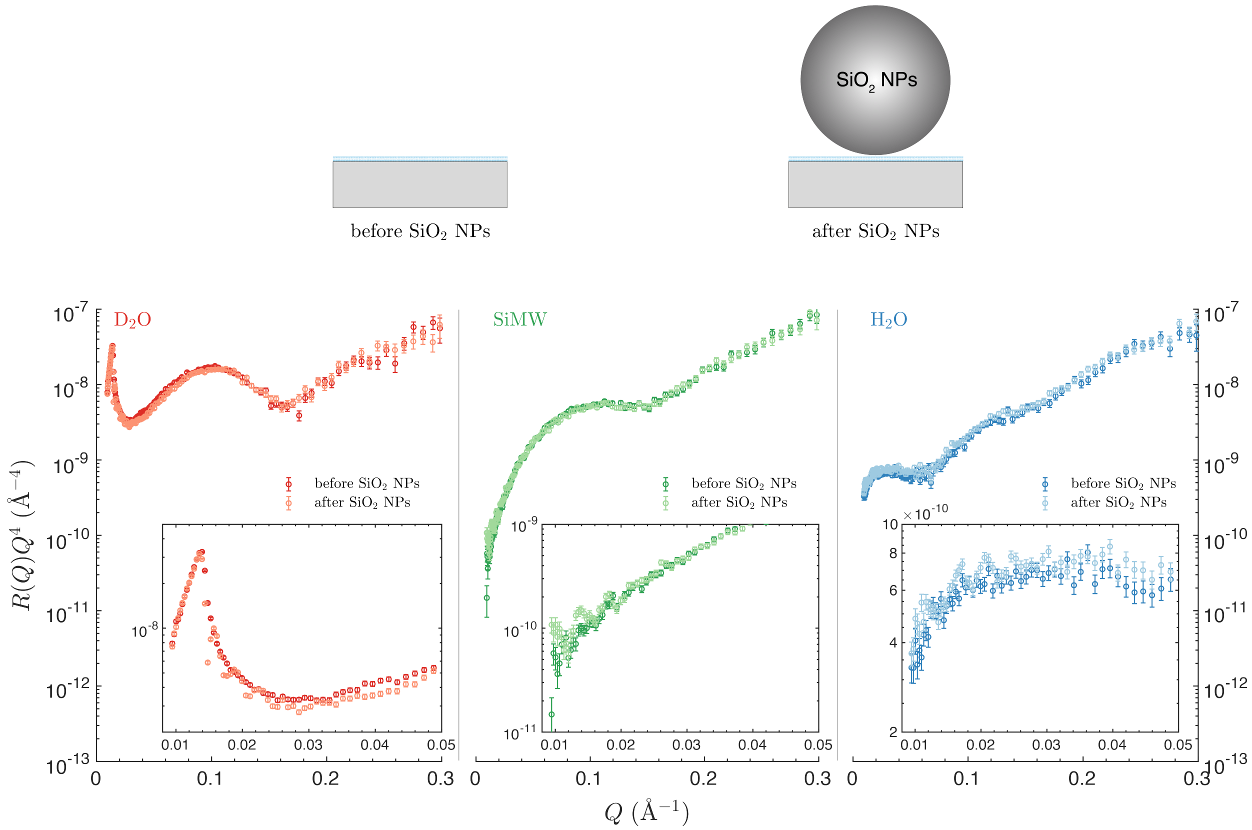

Figure S15: **NR data before and after the SiO_2 NPs adsorption step.** The presence of high-frequency fringes in the low Q range in D_2O, SiMW, and H_2O indicates the formation of a thick layer. However, the overall shape of the whole reflectivity curves remained unchanged, indicating that no distortion, e.g., rapping or lifting, of the planar SLB occurred due to the adsorption of the SiO_2 NPs.

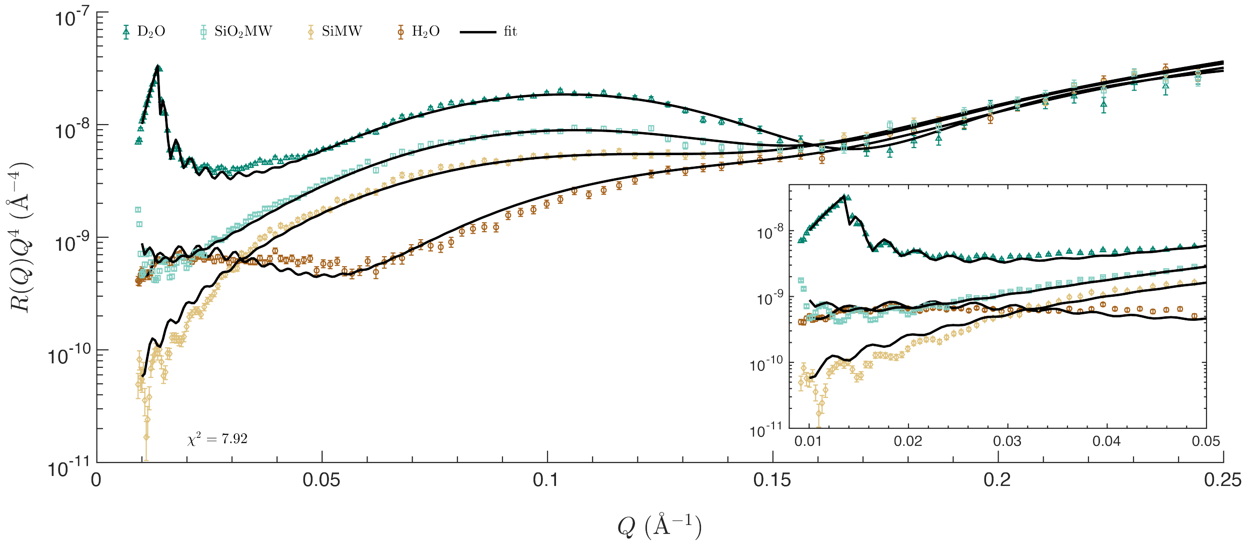

Figure S16: **Example for fitting the nanoSLBs using a one-component particle.** NR data and fits for ABCD-attached nanoSLBs, assuming they are homogeneous (single SLD) with no distinction between the core and the shell. The disagreement between model and experimental data demonstrates the significance of the lipid shell contribution to the NR signal.

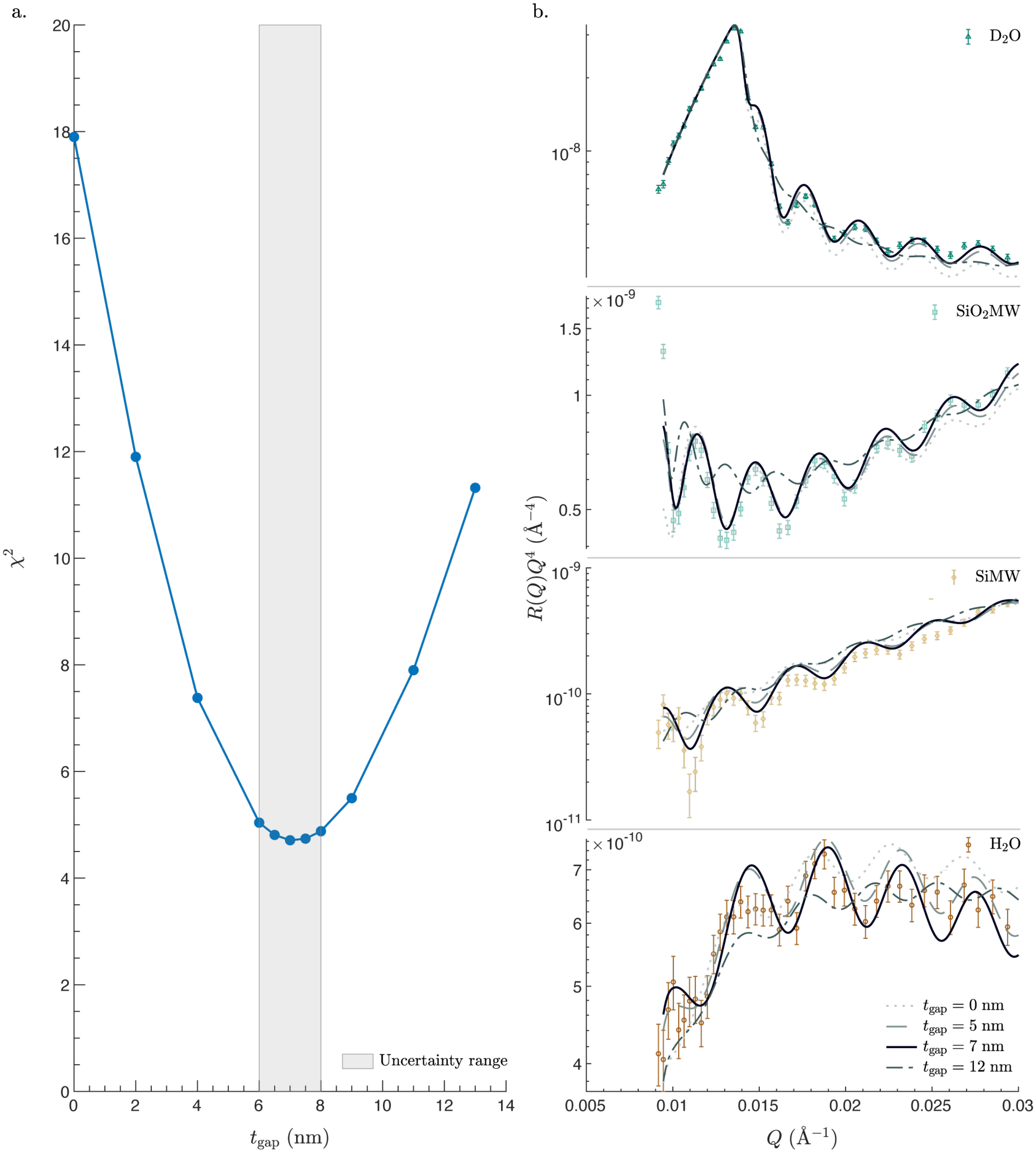

Figure S17: **Effect of t_{gap} on χ^2 and data fitting.** (a) χ^2 versus t_{gap} for the ABCD-nanoSLBs layer shows how changing t_{gap} by as little as 1 nm is enough to result in a substantial increase in the value of χ^2. The shaded area represents the uncertainty range defining the one standard deviation error. (b) Multiple contrasts NR data and fits at different t_{gap} values, with 7 nm represents the best fit with the lowest χ^2. The results demonstrate the sensitivity of the goodness of the fit to any variations in t_{gap}.

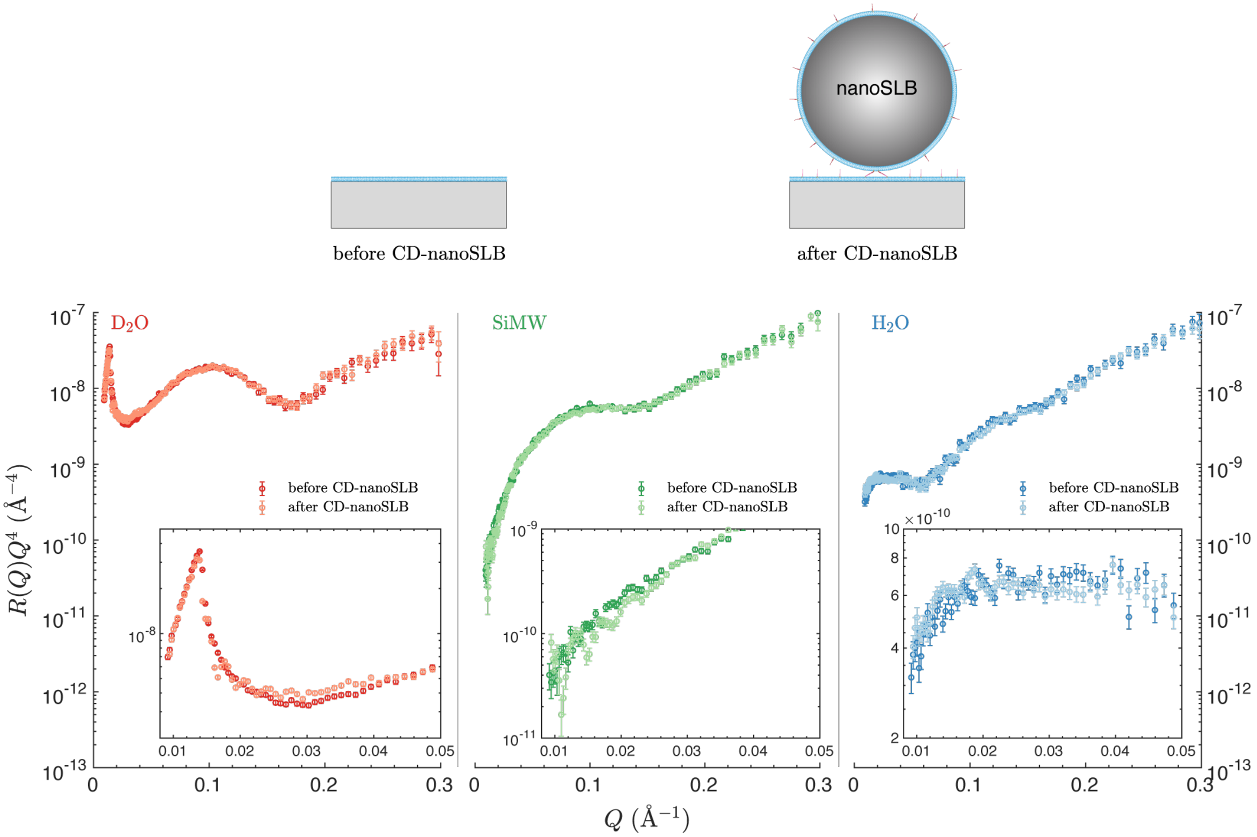

Figure S18: **NR data before and after the CD-nanoSLBs attachment step.** The effect of the CD-nanoSLBs on the NR data is clearly visible, for all investigated contrasts, in the low Q range, where the appearance of high-frequency fringes indicates the formation of a thick layer. The overall shape of the reflectivity curves, particularly in the mid and high Q range, remained, however, unchanged, indicating that no distortion, e.g., rapping or lifting, of the planar SLB occurred due to the attachment of the CD-nanoSLBs.

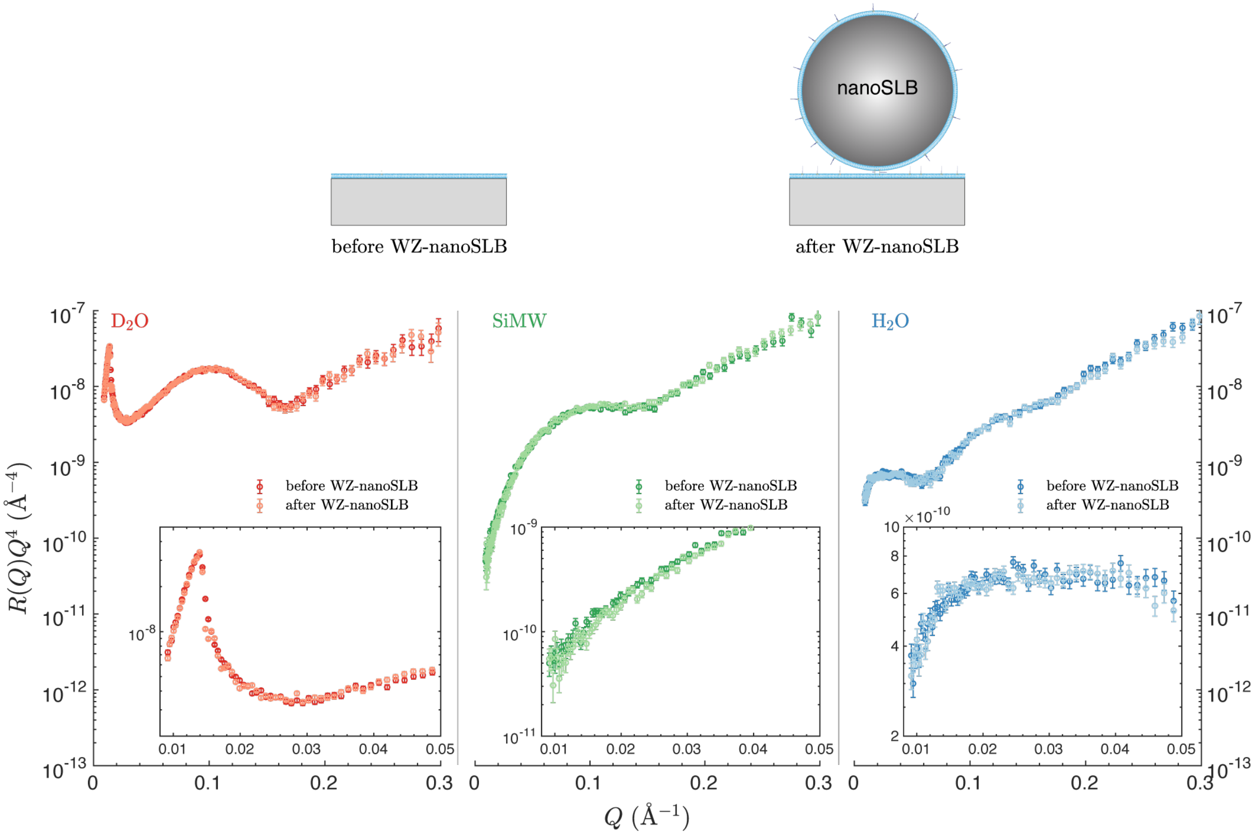

Figure S19: **NR data before and after the WZ-nanoSLBs attachment step.** The effect of the WZ-nanoSLBs on the NR data is less pronounced than the one observed in the case of ABCD-attached nanoSLBs (Figure S18) but still visible at the low Q range where few fringes are present. As for the ABCD-attached nanoSLB samples, the overall shape of the reflectivity curves, particularly in the mid and high Q range, is unaffected by the WZ-nanoSLB addition, indicating that no distortion, e.g., rapping or lifting, of the planar SLB occurred due to the attachment of the WZ-nanoSLBs.

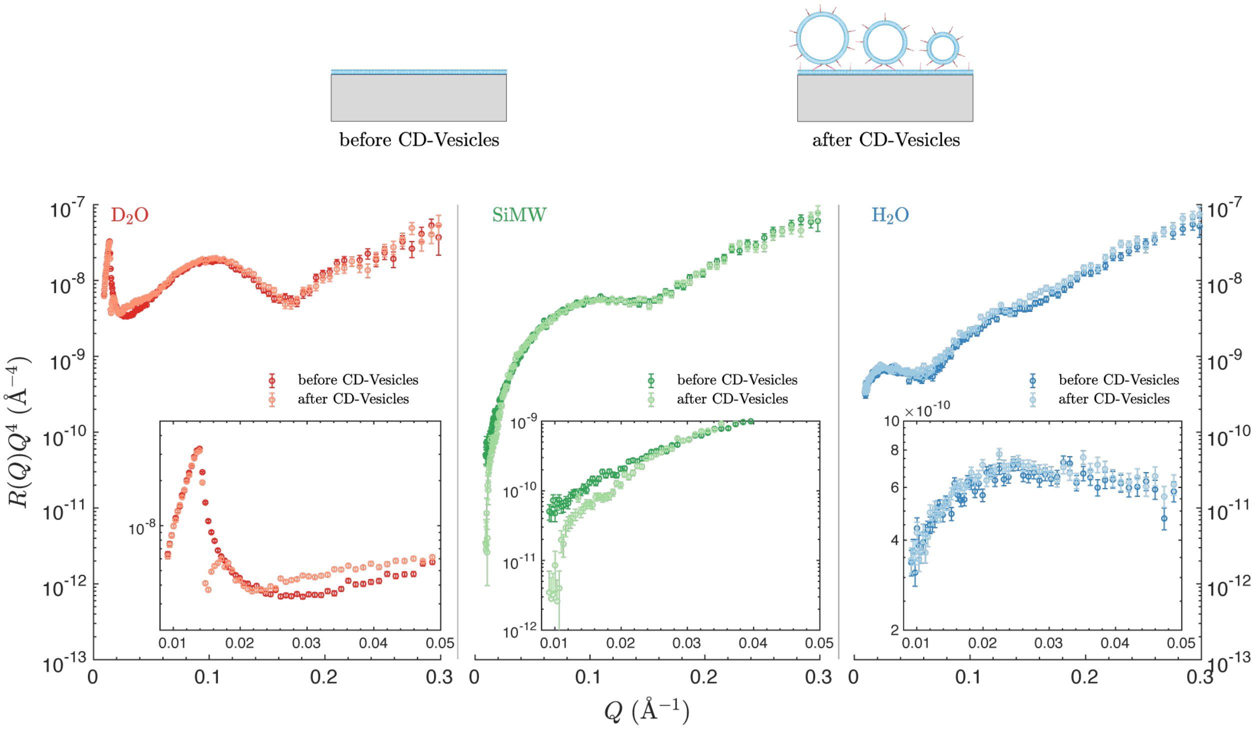

Figure S20: **NR data before and after the CD-vesicles attachment step.** The effect of the CD-vesicles on the NR data is clearly visible for the data measured in D_2O and SiMW solutions, where a large oscillation is present in the low Q range, while it is not visible in H_2O due to the reduced contrast between the vesicles and the solution. The presence of a reduced number of oscillations indicates that the layer is thinner than those observed for SiO_2 NP and nanoSLB samples. The overall shape of the reflectivity curves, particularly in the mid and high Q range, remained, however, unchanged, indicating that no distortion, e.g., rapping or lifting, of the planar SLB occurred due to the attachment of the CD-vesicles.

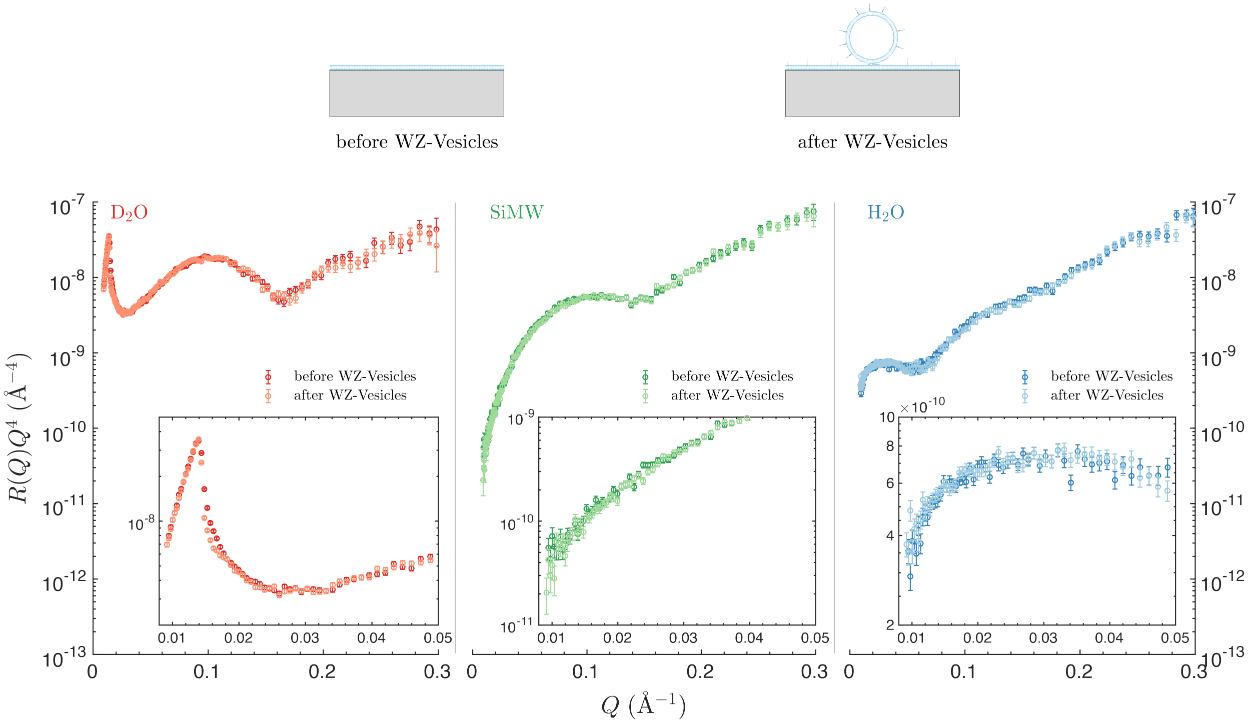

Figure S21: **NR data before and after the WZ-vesicles attachment step.** The effect of the WZ-vesicles on the NR data is visible only for the data measured in D_2O, and it is very small and restricted to the low Q range. Such a change is compatible with the formation of a low-coverage vesicles layer on top of the planar SLB. As for the other samples, the overall shape of the whole reflectivity curves, particularly in the mid and high Q range, remained unchanged, indicating that no distortion, e.g., rapping or lifting, of the planar SLB occurred due to the attachment of the WZ-vesicles.

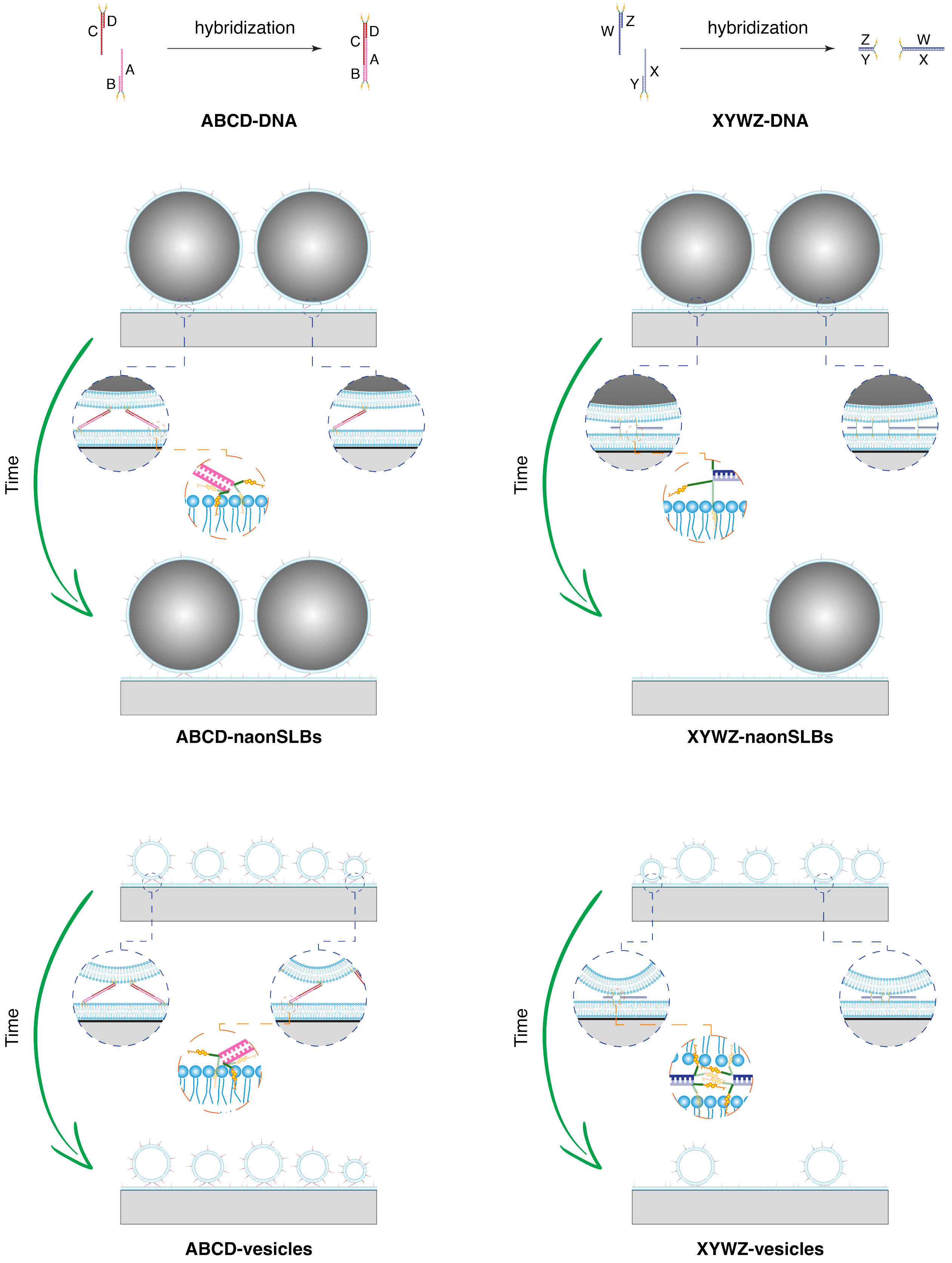

Figure S22: **Effect of ABCD- and XYWZ-DNA on surface coverage and size distribution.** (Continued on the following page.)

Figure S22: **Effect of ABCD- and XYWZ-DNA on surface coverage and size distribution.** Six schematics showing the likely mechanism that underpin the observed higher surface coverage for nanoSLBs and vesicles when immobilized using ABCD-DNA than XYWZ-DNA. The ABCD-DNA differ from the XYWZ-DNA in the configuration by which they tether nanoSLBs and vesicles to the surface. While ABCD-DNA is anchored with two connected cholesterol molecules at both ends of the planar SLB and the nanoSLB/vesicle, the XYWZ-DNA is anchored with only one cholesterol molecule, due to the separation into two linkers upon hybridization of the XY- with the WZ-DNA, forming an XW linker and a YZ linker.[17] It has been previously shown that DNA molecules attached with single cholesterol anchors bind reversibly to lipid bilayers, and those attached with double anchors bind essentially irreversibly.[18,19] This difference is likely due to the continuous switching of cholesterol molecules between the aqueous and lipid phases. Therefore nanoSLBs/vesicles attached via ABCD linkers are expected to remain irreversibly attached; however, nanoSLBs/vesicles attached via XYWZ linkers will only remain attached if there are sufficient number of linkers to keep them attached despite of the continuous switching of cholesterol molecules between the aqueous and lipid phases. Consequently, under the same experimental conditions, it is expected that a lower number of XYWZ-nanoSLBs.vesicles remains attached than ABCD-nanoSLBs/vesicles. For the vesicles, there is an additional factor that smaller vesicles are expected to accommodate smaller number of linkers and thus tend to detach when tethered with XYWZ-DNA, causing a shift in the size distribution of the attached XYWZ-vesicles toward larger sizes.

References

[1] Indriati Pfeiffer and Fredrik Höök. Quantification of oligonucleotide modifications of small unilamellar lipid vesicles. *Analytical Chemistry*, 78(21):7493–7498, 2006.

[2] Antonius Armanious, Yuri Gerelli, Samantha Micciulla, Björn Agnarsson, Hudson Pace, Emanuel Schneck, Rebecca Welbourn, and Fredrik Höök. Exploring the interactions between vesicles/viruses and supported lipid bilayers using neutron reflectometry. *STFC ISIS Neutron and Muon Source*, 2017.

[3] John Webster, Stephen Holt, and Robert Dalgliesh. INTER the chemical interfaces reflectometer on target station 2 at ISIS. *Physica B: Condensed Matter*, 385:1164–1166, 2006.

[4] Yuri Gerelli, Amanda Eriksson Skog, Stephanie Jephthah, Rebecca J L Welbourn, Alexey Klechikov, and Marie Skepo. Spontaneous formation of cushioned model membranes promoted by an intrinsically disordered protein. *Langmuir*, 36:3997–4004, 2020.

[5] O Arnold, J C Bilheux, J M Borreguero, A Buts, S I Campbell, L Chapon, M Doucet, N Draper, R Ferraz Leal, M A Gigg, V E Lynch, A Markvardsen, D J Mikkelson, R L Mikkelson, R Miller, K Palmen, P Parker, G Passos, T G Perring, P F Peterson, S Ren, M A Reuter, A T Savici, J W Taylor, R J Taylor, R Tolchenov, W Zhou, and J Zikovsky. Mantid — data analysis and visualization package for neutron scattering and μ SR experiments. *Nuclear Instruments and Methods in Physics Research A*, 764:156–166, 2014.

[6] Yuri Gerelli. Aurore: new software for neutron reflectivity data analysis. *Journal of Applied Crystallography*, 49:330–339, 2016.

[7] T L Crowley, E M Lee, E A Simister, and R K Thomas. The use of contrast variation in the specular reflection of neutrons from interfaces. *Physica B: Condensed Matter*, 173:143–156, 1991.

[8] Fred James. *MINUIT – function minimization and error analysis reference manual.* Cern, cernlib-d5 edition, 1998.

[9] Norbert Kučerka, Mu-ping Nieh, and John Katsaras. Fluid phase lipid Areas and bilayer thicknesses of commonly used phosphatidylcholines as a function of temperature. *Biochimica et Biophysica Acta*, 1808:2761–2771, 2011.

[10] Shirin Nouhi, Marc Pascual, Maja S. Hellsing, Habauka M. Kwaambwa, Maximilian W.A. Skoda, Fredrik Höök, and Adrian R. Rennie. Sticking particles to solid surfaces using moringa oleifera proteins as a glue. *Colloids and Surfaces B: Biointerfaces*, 168:68–75, 2018.

[11] F. A. L. Dullien. *Porous media fluid transport and pore structure.* Academic Press, New York, 1992.

[12] Antonius Armanious, Björn Agnarsson, Anders Lundgren, Vladimir P. Zhdanov, and Fredrik Höök. Determination of nano-sized adsorbate mass in solution using mechanical resonators: elimination of the so far inseparable liquid contribution. *Journal of Physical Chemistry C*, 125:22733–22746, 2020.

[13] Eric Weisstein. Spherical segment from MathWorld: A Wolfram web resource., 2022.

[14] Michael Kotlarchyk and Sow-Hsin Chen. Analysis of small angle neutron scattering spectra from polydisperse interacting colloids. *The Journal of Chemical Physics*, 79(5):2461–2469, 1983.

[15] Joseph C Fogarty, Mihir Arjunwadkar, Sagar A Pandit, and Jianjun Pan. Atomically detailed lipid bilayer models for the interpretation of small angle neutron and X-ray scattering data. *Biochimica et Biophysica Acta*, 1848(2):662–672, 2015.

[16] Barbara Eicher, Frederick A Heberle, Drew Marquardt, N Gerald, John Katsaras, and Georg Pabst. Joint small-angle X-ray and neutron scattering data analysis of asymmetric lipid vesicles. *Journal of Applied Crystallography*, 50:419–429, 2017.

[17] Gudrun Stengel, Raphael Zahn, and Fredrik Höök. DNA-induced programmable fusion of phospholipid vesicles. *Journal of the American Chemical Society*, 129(31):9584–9585, 2007.

[18] Stef A. J. van der Meulen, Galina V. Dubacheva, Marileen Dogterom, Ralf P. Richter, and Mirjam E. Leunissen. Quartz crystal microbalance with dissipation monitoring and spectroscopic ellipsometry measurements of the phospholipid bilayer anchoring stability and kinetics of hydrophobically modified DNA oligonucleotides. *Langmuir*, 30(22):6525–6533, jun 2014.

[19] Indriati Pfeiffer and Fredrik Höök. Bivalent cholesterol-based coupling of oligonucletides to lipid membrane assemblies. *Journal of the American Chemical Society*, 126:10224–10225, 2004.
